# Beta‐Radiation‐Resistant Anticorrosion Coatings Based on Lignin

**DOI:** 10.1002/smsc.202500007

**Published:** 2025-06-30

**Authors:** Ievgen Pylypchuk, Oleg Tkachenko, Tetyana Budnyak, Mika Sipponen

**Affiliations:** ^1^ Department of Materials and Environmental Chemistry Stockholm University Svante Arrhenius väg 16C 10691 Stockholm Sweden; ^2^ Division of Nanotechnology and Functional Materials Department of Materials Science and Engineering Uppsala University The Ångström Laboratory, Lägerhyddsvägen 1 751 03 Uppsala Sweden; ^3^ Department of Earth Sciences Uppsala University Villavägen 16 752 36 Uppsala Sweden; ^4^ Wallenberg Initiative Materials Science for Sustainability Department of Earth Sciences Uppsala University Villavägen 16 752 36 Uppsala Sweden; ^5^ Wallenberg Wood Science Center Department of Materials and Environmental Chemistry Stockholm University SE‐10691 Stockholm Sweden

**Keywords:** anticorrosion, beta‐particles, coatings, copper, lignin, radiation

## Abstract

As humanity ventures beyond Earth, developing radiation‐stable coatings from non‐fossil sources becomes essential. Beta radiation can significantly harm materials, making it essential to seek resilient, biobased alternatives to work in corrosive environments and high temperatures. Herein, a novel lignin‐based coating demonstrating exceptional beta‐radiation resistance and anticorrosion properties is presented. The coatings are applied to copper substrates and exposed to 500 kGy electron beam irradiation in air to evaluate their structural and functional stability under extreme conditions. Spectroscopic, microscopic, and thermogravimetric analyses confirm the structural integrity of the coatings post‐irradiation. Anticorrosion efficiencies after irradiation are maintained at 99.6% (H_2_SO_4_) and 99.8% (NaCl) for 61 μm thick films, while thinner 9.5 μm films show 86.4% and 85.7% protection in the respective media, with a ≈4% performance drop post‐irradiation. Adhesion strength improves from 0.28 to 0.49 MPa after irradiation, and the water contact angle decreases from 74° to 66°, indicating an increase in hydrophilicity. The superior performance is attributed to the aromatic structure of lignin and its thermally triggered cyclization, which renders it stable against chemical chain scission by oxygen radicals formed in atmospheric conditions under radiation exposure. The performance of thicker films in anticorrosion tests is attributed to a reduced penetration of corrosive agents, due to better morphological integrity. These findings demonstrate the viability of lignin‐based coatings as radiation‐stable and environmentally sustainable solutions for protecting metal surfaces in harsh environments.

## Graphical abstract

Lignin‐based anticorrosion coatings are remarkable in their resistance to beta radiation, maintaining their structural integrity even when other natural polymers break down. This study illustrates this by showing a man traveling beyond Earth in a sauna‐like spaceship, symbolizing durability in extreme conditions. It highlights lignin's potential for creating sustainable, radiation‐resistant materials for industries operating in harsh environments.

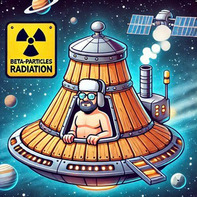

## 1 Introduction

Corrosion is a significant problem in industries such as oil and gas, aerospace, and marine due to its detrimental effects on metal substrates. In extreme environments such as elevated temperatures, high salinity, and radiation sources, the choice of anticorrosion coatings for metal becomes crucial. For instance, coatings with high thermal stability and excellent adhesion to metal substrates are needed under high temperature conditions. Similarly, coatings with excellent barrier properties and oxidative resistance are required in environments with high salinity. Under radiation sources, coatings with radiation resistance properties are necessary to prevent the degradation of the metal surface. There is no doubt that combining all these protection modalities in one material presents a formidable challenge.

Recent advances in sustainable anticorrosion coatings have shown promising results in protecting metals under harsh environments such as elevated temperature,^[^ ^1^ ^]^ high salinity, and radiation sources.^[^ ^2^ ^]^ Still, materials used for anticorrosion coatings, such as polyether ether ketone (PEEK),^[^ ^3^ ^]^ organosilicon,^[^ ^1^ ^]^ and polyurethane (PU),^[^ ^4^ ^]^ are derived from fossil resources. For instance, PU coatings are widely used due to their excellent mechanical properties, corrosion resistance, and versatility, and are widely reported in the literature.^[^ ^5^, ^6^, ^7^ ^]^ To address sustainability issues, researchers are exploring the use of biobased materials and coatings derived from renewable resources.^[^ ^8^ ^]^

Lignin, an abundant and renewable aromatic macromolecule, can be a promising alternative in PU formulations. For example, lignin‐based polyols synthesized via thiol‐ene chemistry^[^ ^6^ ^]^ have been shown to impart comparable anticorrosive properties while improving thermal stability and introducing a biobased content of over 40%. Positive environmental impacts are obvious merits of biobased anticorrosion coatings, especially based on abundant and renewable lignocellulosic resources. Substantial attention has been recently paid to lignin nanoparticles due to their dispersibility in water, which has led to numerous promising applications, including water‐based adhesives,^[^ ^7^ ^]^ hydrogen peroxide sensors,^[^ ^8^ ^]^ and particulate coatings for aluminum^[^ ^9^ ^]^ and steel protection.^[^ ^10^, ^11^, ^12^, ^13^ ^]^

Recent advancements in radiation‐resistant coatings have focused on enhancing the durability and functionality of materials exposed to high‐radiation environments such as nuclear reactors, aerospace systems, and sensing devices. Key innovations include developing nanocomposite polymer coatings that exhibit improved resistance to corrosion, thermal degradation, and mechanical wear, along with self‐healing and antifouling capabilities.^[^ ^14^, ^15^ ^]^ Metal and ceramic‐based systems, such as electroless nickel–boron and SiC/SiC composites with functional overcoats like tungsten, have shown remarkable stability under neutron irradiation and high‐temperature conditions.^[^ ^16^, ^17^ ^]^ Additionally, radiation‐cured coatings based on UV‐curable silicone and urethane acrylates have demonstrated strong adhesion and abrasion resistance.^[^ ^18^ ^]^ In contrast, specialized coatings for optical fibers maintain mechanical integrity at temperature up to 400 °C under high radiation doses.^[^ ^19^ ^]^ Despite these advancements, there remains a significant gap in developing biobased radiation‐resistant coatings, as most organic materials are inherently vulnerable to radiation‐induced degradation. Consequently, current efforts have largely focused on inorganic or synthetic polymer systems.

Protecting copper in radiation‐exposed environments like nuclear waste storage, where it is commonly used, offers a relevant test case for lignin‐based coatings. For instance, in Sweden, the government approved using copper canisters for the disposal of spent nuclear fuel^[^ ^20^ ^]^ by placing them underground. This is particularly important for maintaining the integrity of copper containers in underground deposits. Constant radiation loads can affect containers in contact with soil, causing depressurization and undesired environmental issues. This highlights the importance of developing anticorrosion coatings for copper that do not compromise its performance under radiation exposure. To advance sustainability, it would be beneficial to create such coatings from biobased and renewable resources.

In this context, lignin is radiation‐stable, meaning it does not change its chemical structure upon a radiation dose of 200 kGy.^[^ ^21^ ^]^ Therefore, lignin can be a plausible candidate due to its radiation stability, abundance, and presence of phenolic hydroxyl units, which act as antioxidants. Additionally, its aromatic nature potentially provides it with improved thermal stability.^[^ ^22^ ^]^

Considering all the aforementioned factors, we hypothesized that introducing thermally curable chemical functionalities into the lignin macromolecule would enable the development of radiation‐resistant anticorrosion coatings. We show through electrochemical, spectroscopic, microscopic, and thermogravimetric tests that such lignin‐based advanced coatings provide competitive protection of copper under harsh conditions.

## 2 Results and Discussion

### 2.1 Development of the Coating System

Protection of copper against corrosion and beta radiation is essential, owing to the advanced engineering solutions in which it is used. Our approach was to use lignin as a natural polyphenol with known radical scavenging properties^[^ ^23^ ^]^ to develop protective coatings for copper. We reasoned that activating lignin by introducing ‐yne functionalities would be a plausible route to cured products, as copper can catalyze reactions involving triple carbon bonds, such as yne‐azide click reactions.^[^ ^24^ ^]^ As shown in many examples, Cu can also play a role in triple bond thermal polymerization, including chain extension reactions of propargylated lignin^[^ ^25^ ^]^ (Pro‐lignin).

**Figure 1:**
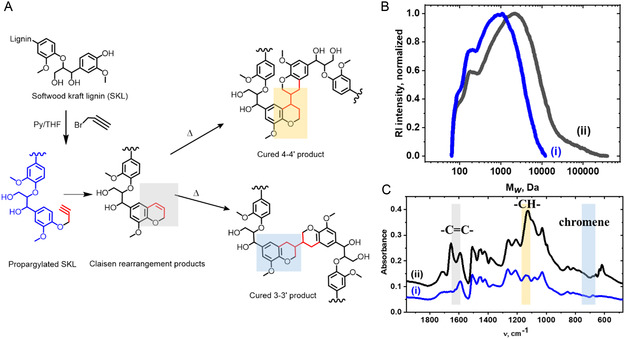
A) Suggested curing mechanism for the pro‐lignin; B) SEC elution profile, and C) FTIR spectra for (i) the initial and (ii) cured propargylated lignin.

After the propargylation, the lignin with phenolic OH substitution degree 46% (^31^P NMR, Figure S1, Supporting Information; ^1^H NMR Figure S2, Supporting Information) was dissolved in DMF and subjected to self‐curing at 150 °C for 4 h, as shown in **Figure** **1**A. Due to the high reactivity of the propargyl groups, an increase in molecular weight (M_*w*_) due to polymerization was observed already after 1 h at 105 °C (Figure 1B).

Heating of propargylated lignin in DMF at 150 °C leads to its self‐curing due to several chemical reactions and suggests a pathway to cyclic chromene structures from propargylated softwood kraft lignin, due to Claisen rearrangement, as shown in Figure 1A. These cyclic structures can be identified by a Fourier transform infrared spectrometer (FTIR) for thermally cured samples at 1650, 1127, and 637 cm^−1^ in Figure 1C, which correspond respectively to absorption bands of newly formed double bonds due to Claisen rearrangement, aliphatic bonds, and the chromenes, determined earlier.^[^ ^26^ ^]^

Chemical cyclization provides greater molecular stability at higher temperatures in an oxygen atmosphere since cyclic molecular motifs hinder radical propagation. For example, the formation of thermally stable cyclic aromatic polymers is known for benzoxazines,^[^ ^27^, ^28^ ^]^ improving their flame‐retardancy.^[^ ^29^ ^]^ A similar thermal behavior has been observed for the cyclic lignin polymer obtained in this study. Elevated thermal stability due to cyclic molecular motif rendering cured products with fire‐retardant properties can be observed in supplementary Video 1.

**Figure 2:**
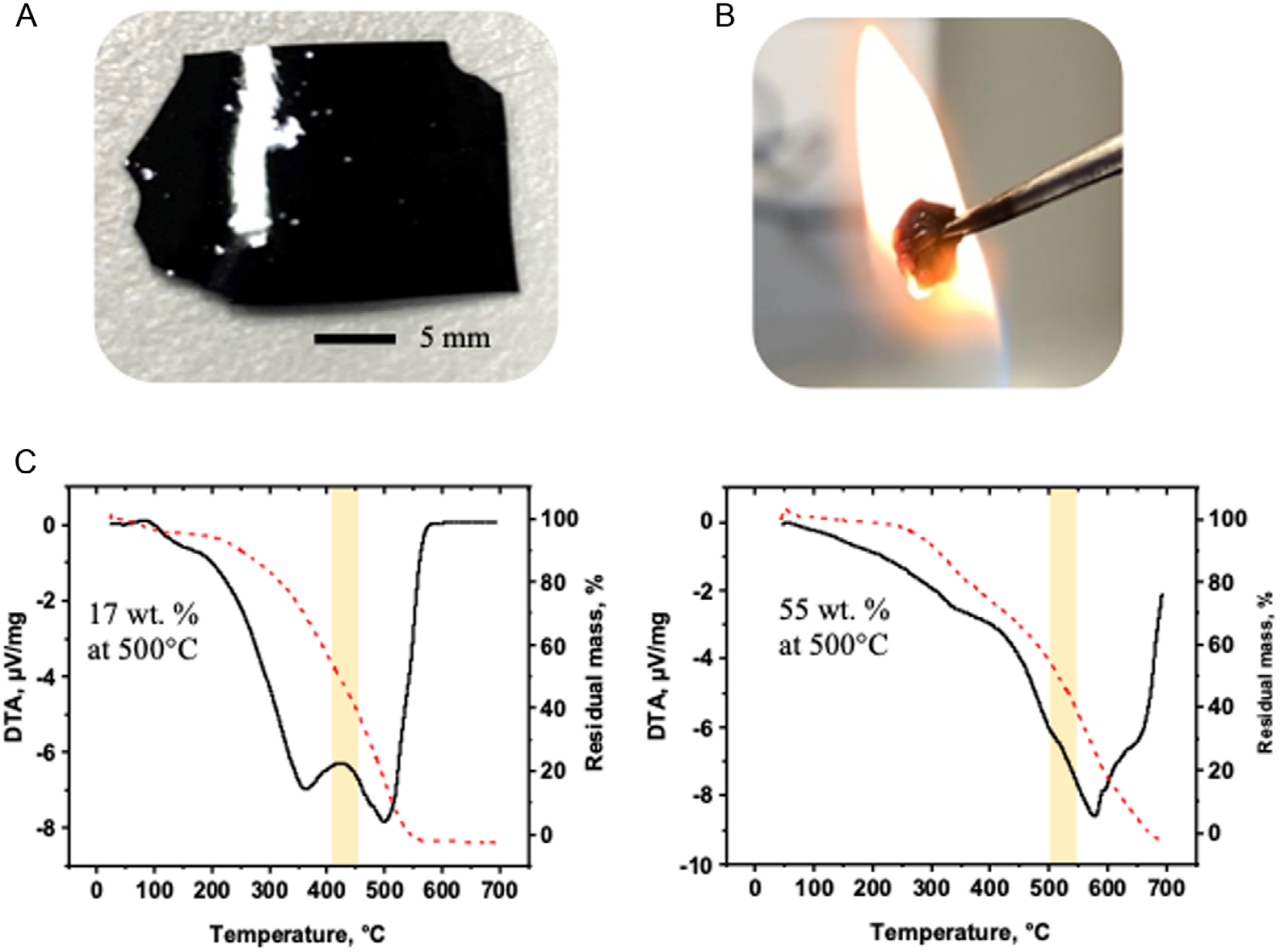
A) Visual appearance and B) a snapshot of the cured film exposed to open flame, and C) thermogravimetric data for the initial Pro‐Lignin and cured film.

The resulting curing product is a freestanding plastic‐like material with a glossy black appearance, as depicted in **Figure** **2**A. This cured lignin product was thermally stable and resistant to open fire (Figure 2B). According to DTA‐TGA, the polymerization product has better thermal stability in the air than the initial Pro‐lignin, as shown in Figure 2C. The cured product maintains 55% of its weight at 500 °C, while the initial Pro‐lignin has only 17% of its initial weight at this temperature (thermogravimetric analysis (TGA) analysis in Figure 2C). Moreover, at 550 °C, uncured Pro‐lignin is completely burned, while the cured product keeps 36% of its original weight. In this context, the improved thermal stability in the air led us to consider various applications for such material, such as organic coating for metal protection. However, the cured free‐standing lignin product turned out to be brittle. Brittleness can make materials susceptible to cracking and fracture under stress or deformation.

**Figure 3:**
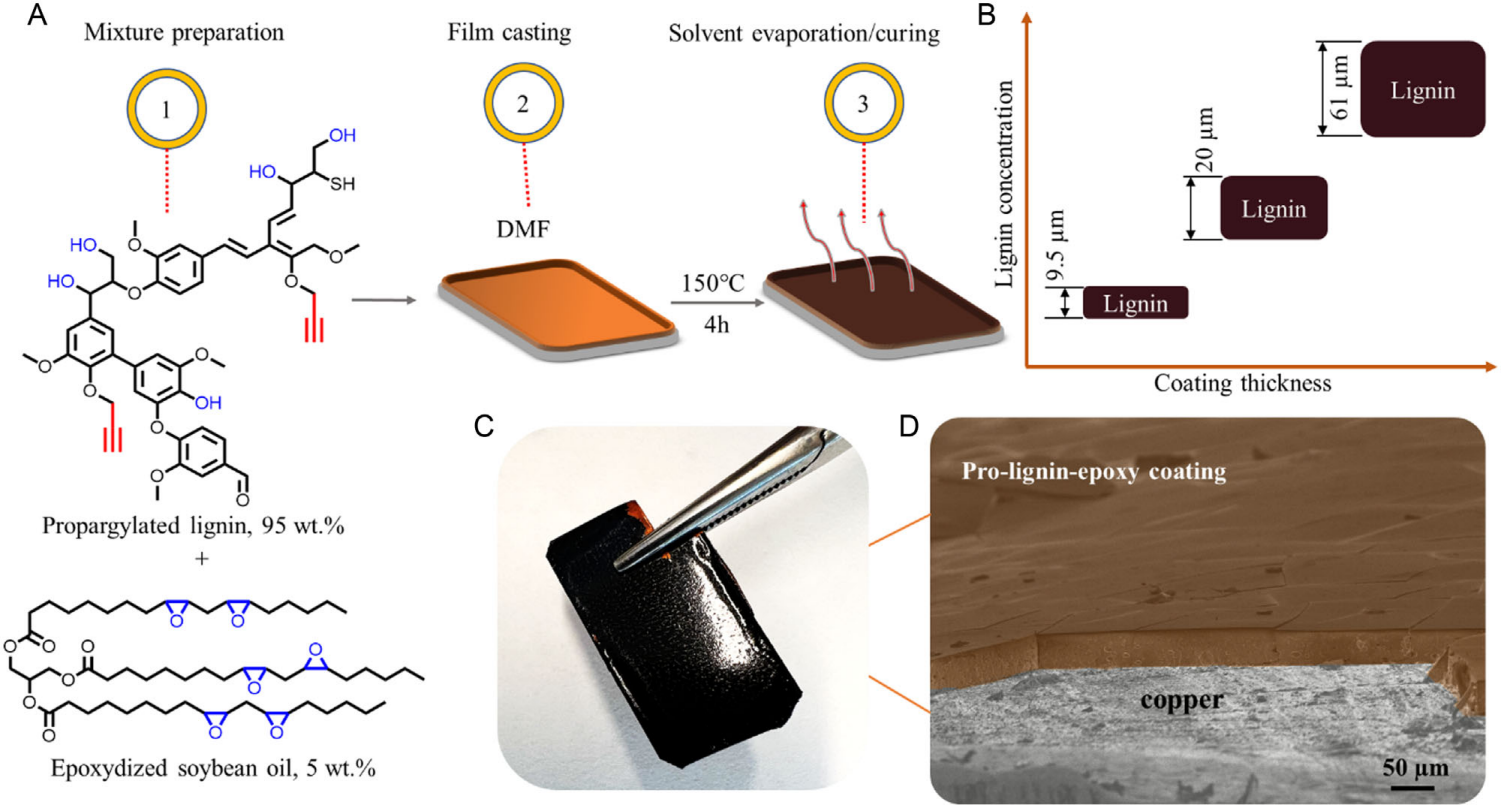
A) Scheme for the preparation of lignin coating; B) samples thickness as a function of lignin concentration; C) visual appearance of the coating; and D) SEM micrograph of the coating with the brown phase showing the false‐colored coating layer.

To address the brittleness of the freestanding material, we incorporated 5 wt% of epoxy soybean oil as a plasticizer (**Figure** **3**A). Such solutions were cast over the Cu surface and thermally cured, as shown in the figure below. In addition, we tested different lignin concentration and their effect on the coating thickness. An increased concentration led to an increase in the coating's thickness, as shown in Figure 3B. Scanning electron microscopy (SEM) has confirmed the visual appearance of such a coalition and the formation of a continuous layer (Figure 3C,D, respectively).

### 2.2 Anticorrosion Efficiency

**Figure 4:**
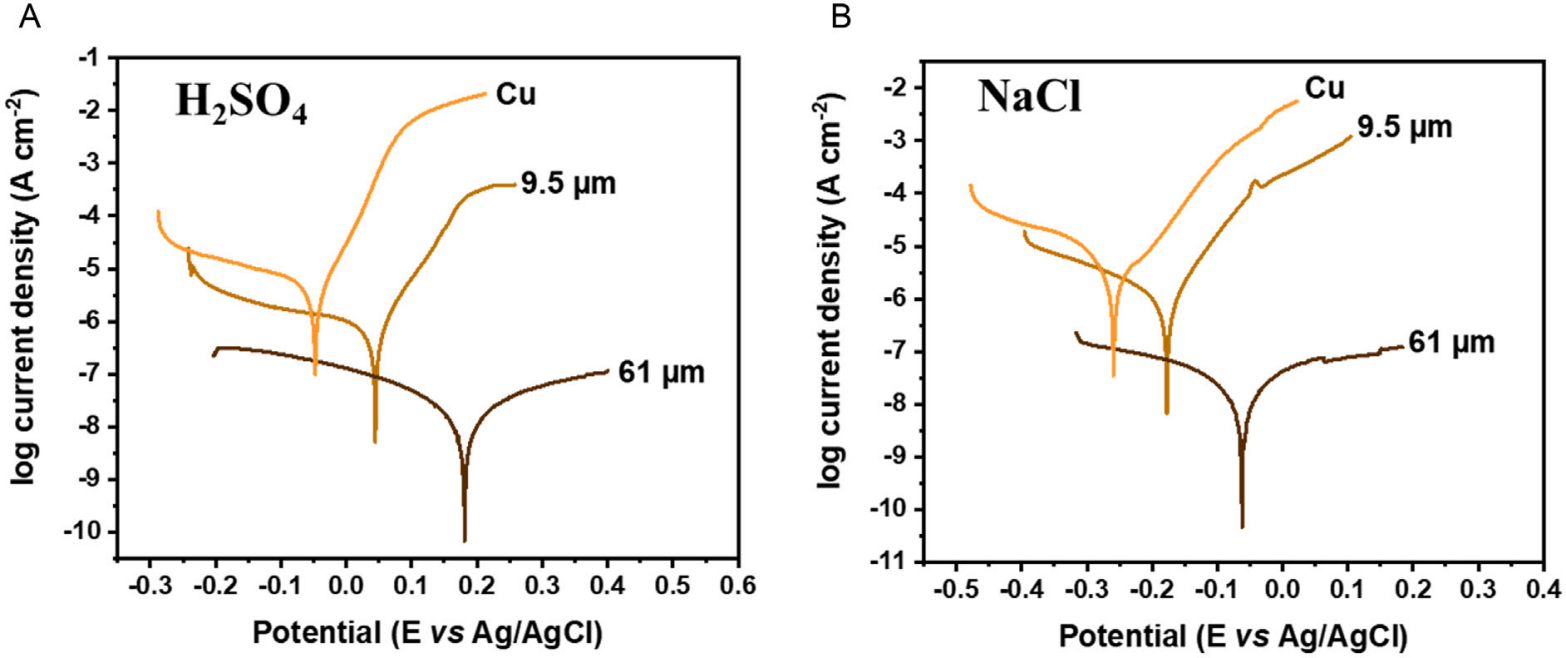
Tafel plots showing anticorrosion properties of Pro‐lignin coating in A) 0.5 M H_2_SO_4_ and B) 3.5% NaCl.

Considering the ability of the developed lignin‐based thermosetting formulation to form a continuous film with good adherence to the copper surface, a coating was then investigated using the polarization technique. The electrochemical properties and anticorrosion efficiency were examined in 3.5% NaCl and 0.5 M H_2_SO_4_ solutions. **Figure** **4**A demonstrates the polarization curves in Tafel extrapolation of the protected and bare copper electrodes immersed in corrosive solutions. As expected, the cathode and anode current densities of the protected Cu electrodes are much lower than those of the bare Cu, indicating the effective inhibition of the corrosion process. Equation (1) and (2) were applied to the Tafel plots to quantify the efficiency of the protective films based on lignin. The obtained parameters are listed in Table S1, Supporting Information.

The Pro‐lignin coating (no epoxy added) demonstrated 60% protective efficiency after 24 h of immersion in 3.5% NaCl. However, the film was not stable in 0.5 M H_2_SO_4_ solutions. SEM micrographs (Figure S3, Supporting Information) show a visually homogeneous coating with a c.a. 10 μm thickness and several microscopic defects. The surface morphology of the Pro‐lignin coating revealed the presence of separated mountain‐shaped islands across the surface, which allow film penetration by hydrated hydronium ions, destroying the film. The preparation of a more uniform coating without defects would enhance the protection of the copper surface from corrosive media. To prevent exposure of copper to the corrosive environment, we searched for ways to improve the integrity of the coating. Since lowering the curing temperature to 110–120 °C did not produce a notable improvement (data not shown), we reasoned that the problem lies in the original stiffness of the lignin molecule, which is anticipated to increase due to the curing reaction and result in the brittle coating. Simply adding PEG‐400 to the curing composition improved the smoothness of the coating as observed in SEM (Figure S4a, Supporting Information), although it did not enhance anticorrosion efficiency due to the washing out effect after immersion in aqueous solutions of both corrosive agents overnight (Figure S4b, Supporting Information).

We added a low‐M_*w*_ molecule that can co‐cure and serve as a flexible chain linker to overcome the cracking and ensure improved water stability. Soybean epoxy oil was chosen due to its natural source and industrial availability. The proposed curing reaction is presented in Figure S5, Supporting Information. As a result of the addition, higher coating integrity (sample *lignin 1x*) translated to improved film stability and anticorrosion protection in salt and acid media (Figure 4A,B). The protective efficiency reached 86% for both media (Table S1, Supporting Information). As can be observed from the SEM image (Figure S6, Supporting Information), the formed coating is homogeneous and thinner compared to the coating without the electron micrograph, which shows that this coating did not develop cracks during 24 h of immersion in water. The obtained Pro‐lignin epoxy demonstrated high resistance to water but still had relatively low protective efficiency; therefore, we increased the film thickness to improve its anticorrosion behavior. Almost 100% protection (more details in Table S1, Supporting Information) was achieved when the thickness became 61 μm (sample *lignin 4x*) instead of 9.5 μm observed for the *lignin 1x* coating.

### 2.3 Anticorrosion Properties After Radiation Exposure

**Figure 5:**
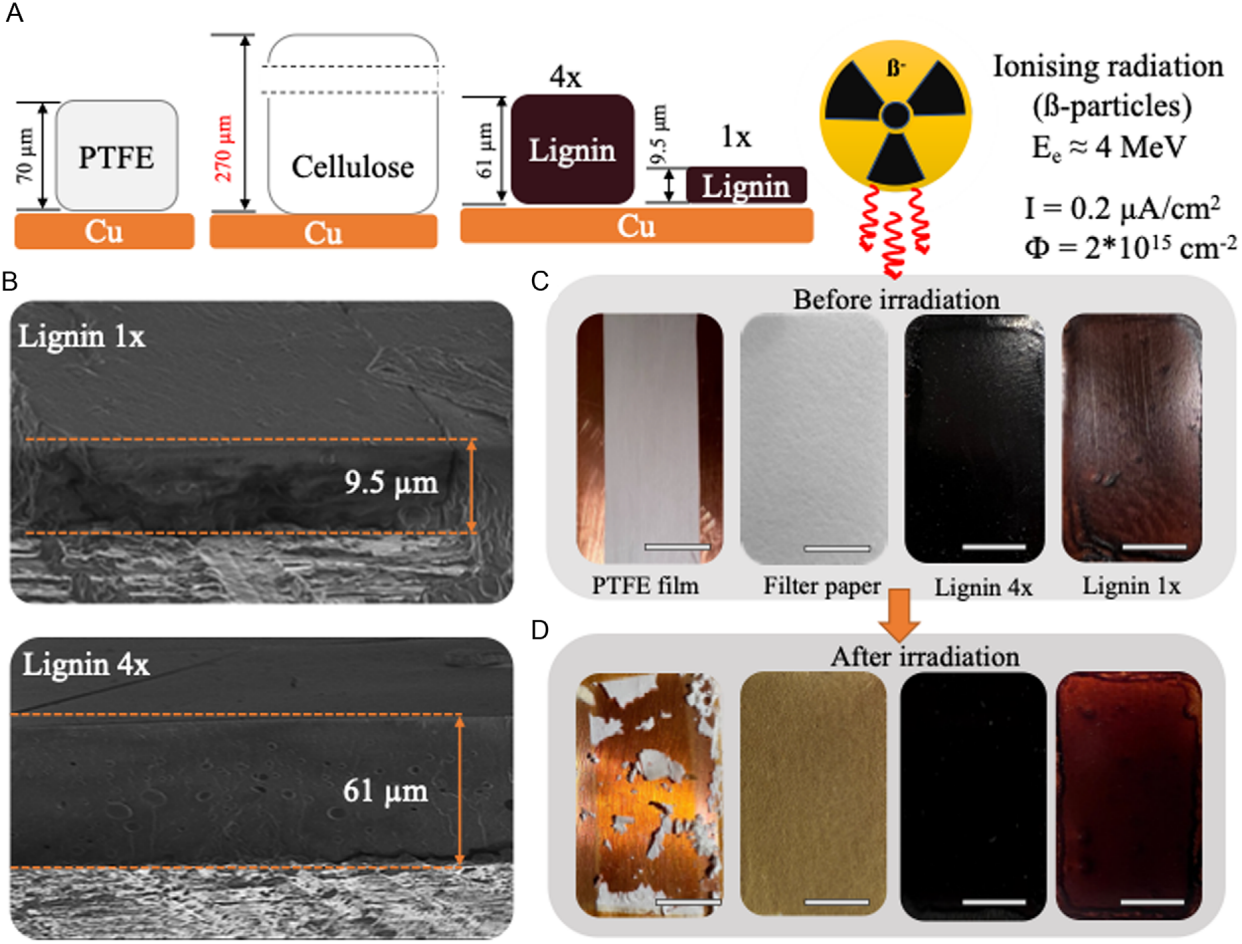
A) Scheme for samples and their thickness; B) cross section image of irradiated lignin samples with different thicknesses; C) visual appearance of initial lignin and reference samples; and D) visual appearance of irradiated samples. Scale bar = 1 cm.

An epoxy‐containing lignin coating was used for the radiation tests due to its higher integrity and better corrosion protection properties. The coatings, with different thicknesses and reference materials, were exposed to beta‐particle irradiation (an electron beam with energy 4 MeV) in an air atmosphere. As reference materials, we used commercially available PTFE (polytetrafluoroethylene) isolation tape and commercial cellulose filter paper with defined thickness (**Figure** **5**A).

SEM micrographs of the lignin coatings with two different thicknesses are shown in Figure 5B. Before‐radiation‐exposure images of reference samples and lignin films with different thicknesses are shown in Figure 5C. To model the potential effect of copper reflecting and re‐emitting photons and electrons under electron beam exposure, reference samples were also placed over the copper piece with the same thickness as for lignin samples.

The high radiation dose caused significant changes in the appearance and integrity of the PTFE tape and the cellulose filter paper (Figure 5D). The PTFE and cellulose (filtering paper) are known to start to decompose after exposure to radiation damage above 50^[^ ^21^ ^]^ and 3.5 kGy^[^ ^30^ ^]^ in air, respectively. In contrast, cured lignin films visually remained intact after the radiation exposure (Figure 5D).

Under exposure to a 4 MeV electron beam, molecular oxygen (O_2_) in air undergoes intense ionization and excitation, initiating a cascade of chemical transformations. Primary interactions with high‐energy electrons produce O_2_^+^ ions and excited O_2_* species, rapidly dissociating into highly reactive atomic oxygen (O·). Due to their unpaired electrons, these oxygen atoms drive the formation of ozone (O_3_). The ozone generation underpins the oxidative environment created by electron beam irradiation, altering the surface of the irradiated coating. The following schemes can describe these processes:

Oxygen ionization:

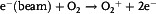




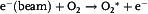

O_2_ *—oxygen in the excited electronic state.

Formation of Atomic Oxygen:

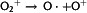




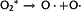




Ozone formation:

$$O\cdot +O_{2}\cdot \to {\text{ O}}_{3}$$

Under radiation exposure in air, PTFE and cellulose undergo significant degradation through different mechanisms. In the case of PTFE, ionizing radiation causes chain scission, resulting in the formation of free radicals that react with oxygen. This reaction leads to the formation of carbonyl and carboxyl groups, resulting in embrittlement and mechanical disintegration of the material. Similarly, in cellulose, radiation generates free radicals that undergo oxidation, resulting in the cleavage of glycosidic bonds and the formation of carbonyl and carboxyl groups. This process depolymerizes, weakens the cellulose structure, and produces volatile degradation products, such as furfural. Additionally, oxidative degradation causes a noticeable color change in cellulose.

**Figure 6:**
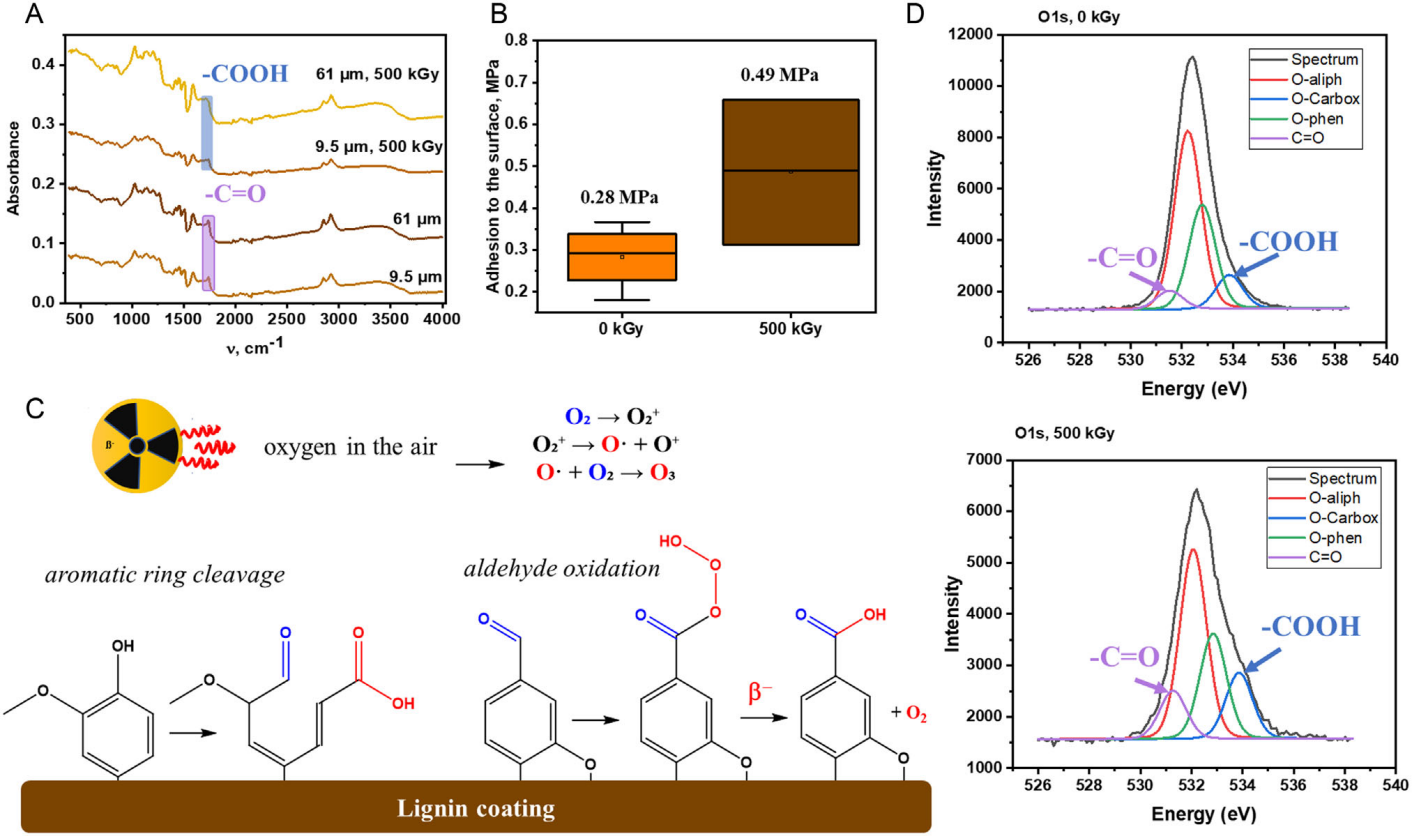
A) FTIR spectra; B) the adhesion strength of the coating to the metal surface before and after irradiation; C) scheme for lignin oxidation under electron beam in air; and D) XPS spectra for lignin coating before (top) and after (bottom) irradiation.

At the same time, after the irradiation, the lignin coating maintains mechanical integrity and appearance. However, the changes were observed in IR spectra and adhesion to the metal surface (**Figure** **6**A,B).

The primary mechanism behind radiation‐induced changes in lignin coating is the ionization of O_2_ molecules in the air. This leads to the formation of reactive oxygen species (ROS), such as O_3_, O_2_^+^, and O_2_^−^. These ionized forms of oxygen attack the coating's surface, oxidizing the lignin. The interaction and oxidation of lignin with ozone have been described in the work.^[^ ^31^ ^]^ The ozone production in the air under an electron beam is detailed in the work.^[^ ^32^ ^]^

Coating exposure to high‐energy radiation in air leads to partial oxidation of lignin, as evidenced by the FTIR and X‐ray photoelectron spectroscopy (XPS) data (Figure 6A,D). After irradiation, the FTIR spectra show an increase in the absorption bands at ≈1740 and ≈1650 cm^−^^1^, corresponding to carbonyl (C=O) and conjugated C=C/C=O structures, respectively. These changes suggest oxidation of aldehydes and unsaturated structures to carboxylic acids and possibly quinones. In particular, the increased intensity of carbonyl‐related bands indicates the formation of new oxygen‐containing groups rather than the disappearance of the carbonyl signal. XPS analysis further supports this interpretation, which reveals a notable 6% increase in the relative surface contribution of carboxylic (–COOH) groups after 500 kGy irradiation (**Table** **1**).

**Table 1:** Change the surface composition of lignin coating before and after irradiation.

Group	0 kGy (%)	500 kGy (%)	Change (Δ%)	Interpretation
O‐aliph	53.25	46.41	↓ −6.84	Decrease suggests cleavage or oxidation of aliphatic C—O bonds
O‐Carbox	9.98	16.14	↑ +6.16	Increase implies formation of carboxylic acids—oxidation product
O‐phenol	31.10	25.67	↓ −5.43	Drop indicates consumption or transformation of phenolic OH (e.g., to quinones or ethers)
C=O	5.67	11.78	↑ +6.11	Significant increase points to oxidation, possibly forming ketones or conjugated aldehydes/quinones

These oxidative modifications correlate with improved coating performance. Pull‐off adhesion testing shows an increase from 0.28 MPa (pristine coating) to 0.49 MPa (irradiated), indicating enhanced interfacial bonding with the copper substrate. We attribute this improvement to the formation of polar functional groups (e.g., carboxyls) and/or changes in microstructure, such as increased surface roughness, which are known to enhance wetting and mechanical interlocking. The proposed mechanism of lignin oxidation is illustrated in Figure 6C.

**Figure 7:**
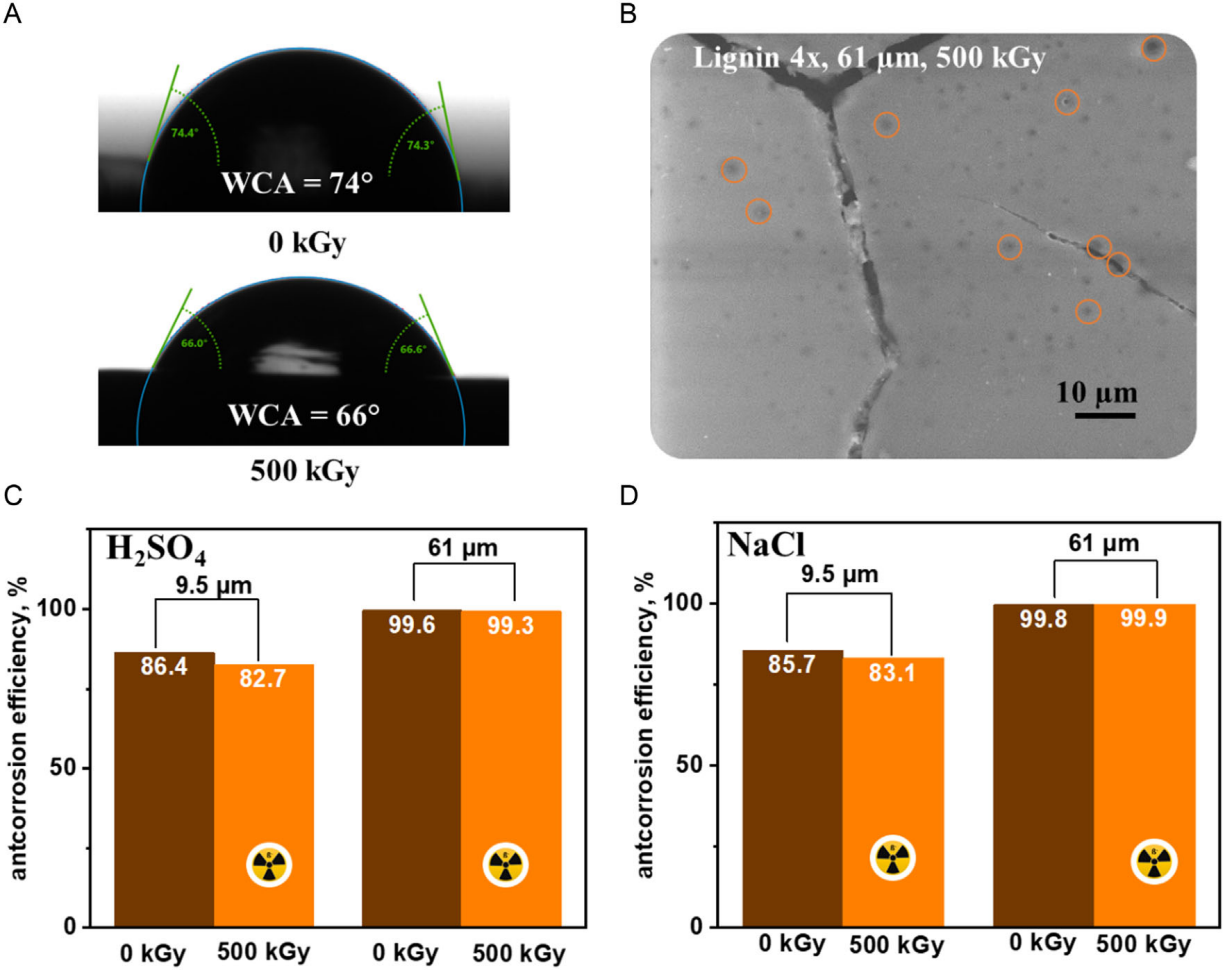
A) WCA of the films before and after irradiation; B) SEM image of the irradiated sample with radiation damage highlighted by red circles; C) Anticorrosion properties of the films, after 24 h immersion in 0.5 M sulphuric acid and D) 3.5% sodium chloride before and after irradiation.

After oxygen activation by a high‐energy electron beam, it reacts with aldehyde groups in lignin, making unstable ozonide intermediates, followed by their transformation to carboxylic ones with oxygen release. The oxidation of the lignin could also be followed by the coating's water contact angle (WCA) measurement (**Figure** **7**A). While the intact coating exhibits around 74°, the WCA changes to 66° after radiation exposure. This we attribute to the formation of the carboxylic groups on the surface, which are known to be more hydrophilic.^[^ ^33^ ^]^ In addition, the SEM observation revealed the presence of dark spots in irradiated samples (Figure 7B). Such spots occur due to the partial oxidative degradation of lignin. Their formation influences the anticorrosion performance of the coating since they may allow penetration of corrosive media to the metal surface.

After the radiation exposure, we measured the anticorrosion efficiencies of lignin coatings and compared them to those of nonirradiated samples. The efficiency of the anticorrosion protection of the films with lignin thicknesses of 9.5 and 61 μm exposed to beta‐particle radiation is presented in Figure 7C and 7D, respectively. Coatings with a layer of 9.5 μm exhibit anticorrosion efficiency towards H_2_SO_4_ and NaCl of 86.4% and 85.7%, respectively. Thicker ones, 61 μm, exhibit anticorrosion efficiency towards H_2_SO_4_ and NaCl of 99.6% and 99.8%, respectively. As can be observed, thinner films (9.5 μm) suffered more from the radiation exposure, losing ≈4% of their protection ability in the corrosive media, dropping to ≈83% of their anticorrosion efficiency.

In contrast, the performance drop for a thicker coating was around 0.3%. The reduced efficiency observed for the thinner (9.5 μm) coatings postradiation, in contrast to the thicker (61 μm) variant, can be primarily attributed to oxygen‐induced degradation processes that occur more readily in thinner films. While it is often assumed that radiation‐induced changes depend solely on the total absorbed dose, this holds predominantly for irradiations conducted in oxygen‐excluded environments or at high dose rates. Under ambient conditions, especially at lower dose rates typical of practical irradiation scenarios, oxidative processes dominate and become inversely related to sample thickness. The surface‐to‐volume ratio is significantly higher in thinner coatings, enabling greater oxygen diffusion throughout the material. This facilitates the formation of ROS under electron beam irradiation, leading to enhanced oxidation of the lignin matrix. As reported in previous studies,^[^ ^34^ ^]^ the radiation stability of polymers under such conditions can drop to as low as 1%–10% of their stability observed at high dose rates or under inert atmospheres. Furthermore, electron beam irradiation in air is particularly aggressive in inducing oxidative degradation compared to gamma irradiation at the same dose.^[^ ^35^ ^]^

Thus, the decreased performance of the 9.5 μm coating can be explained by its increased susceptibility to oxygen‐driven chain scission and oxidative modification, which compromises both the coating's mechanical integrity and protective capacity. In contrast, the 61 μm coating provides a self‐shielding effect, where the inner layers remain relatively protected from oxygen and radical attack, preserving their structural and functional performance postirradiation.

**Table** **2** presents a comparative overview of corrosion protection performance for various biobased coatings, including lignin, chitosan, starch, and cellulose derivatives. Key metrics include electrolyte type, inhibition efficiency (η), and whether the coating was tested under radiation exposure.

**Table 2:** Combined electrochemical comparison of biobased coatings.

Coating system	Electrolyte	η (%), inital	η (%), 500 kGy	Reference
Pro‐lignin epoxy (61 μm)	3.5% NaCl	99.8	99.9	This work
Pro‐lignin epoxy (61 μm)	0.5 M H_2_SO_4_	99.6	99.3	This work
Lignin from black liquor	0.5 M H_2_SO_4_	95	na	[53]
Silanized lignin hybrid	5% NaCl	99	na	[54]
Lignin (solvent‐cast beech)	PBS, pH 7.4	99	na	[55]
Lignin (plasticized) on anodized steel (2‐layer)	3.5% NaCl	97	na	[56]
Chitosan–PTA crosslinked	3.5% NaCl	66	na	[57]
Zn–Eggshell + Starch	3.5% NaCl, 35 °C	94.7	na	[58]
GO–Chitosan–Silver nanocomposite	3.5% NaCl	98	na	[59]
Carboxymethyl Cellulose (CMC)	CO_2_ + 3.5% NaCl	45	na	[60]

Among all coatings, the Pro‐lignin epoxy (61 μm)—both before and after 500 kGy radiation exposure—demonstrated exceptional anticorrosion performance, achieving icorr values as low as 0.00549 μA cm^−2^ and inhibition efficiencies up to 99.94% in 3.5% NaCl. This outperforms even advanced composite coatings such as GO–chitosan–silver (98% η) and silanized lignin hybrids (≈99% η), none of which were tested under radiation.

While thinner, the 9.5 μm Pro‐lignin coatings still showed robust protection (η = 83%–85.7%) and retained most of their efficiency postirradiation, highlighting the importance of film thickness in maintaining oxidative stability. Notably, Pro‐lignin epoxy is the only system in this comparison tested under radiation, which underscores its unique multifunctionality, combining corrosion resistance with resilience under extreme conditions.

Traditional lignin‐based coatings, such as black liquor lignin in acid (η = 95%) or solvent‐cast films in PBS (η = 99%), provide good baseline performance but generally operate under milder or non‐industrial conditions and lack structural durability tuning. Similarly, chitosan and starch‐based systems provide solid mid‐range efficiency (≈66%–95%) but tend to require reinforcement or blending to match the barrier performance of epoxy‐lignin hybrids.

### 2.4 Comparison of Price and Performance to Other Materials

**Figure 8:**
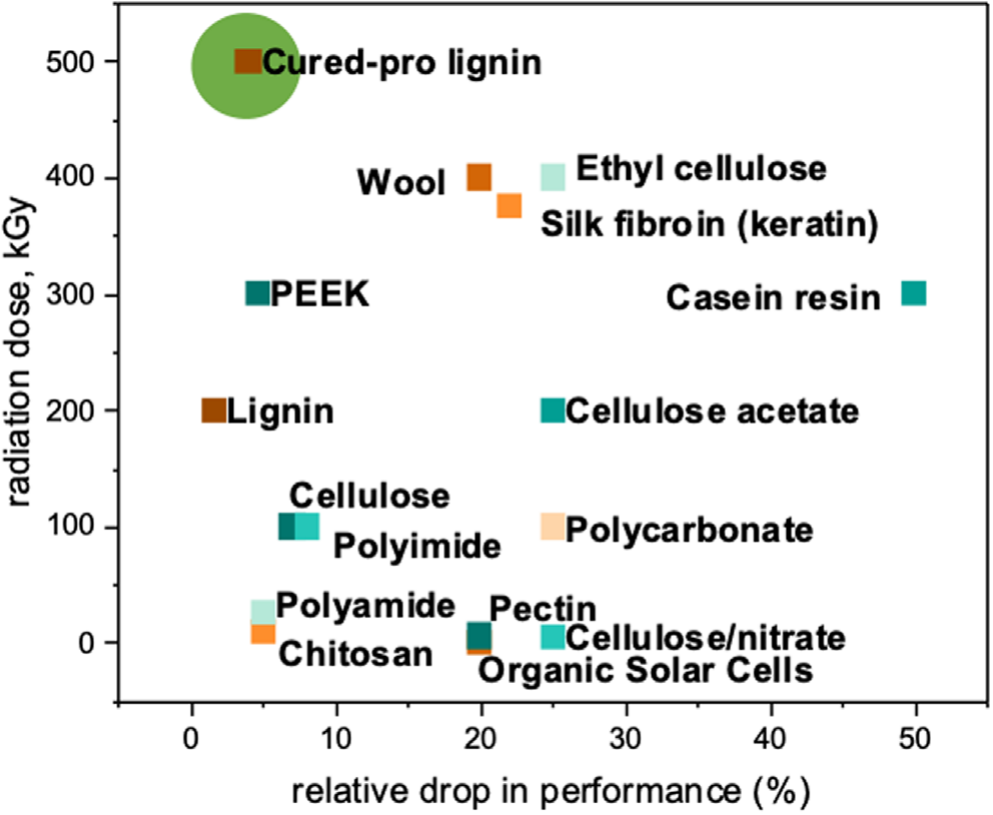
Change in properties of the different materials after radiation exposure.

Lignin demonstrates exceptional radiation stability compared to natural and synthetic polymers, positioning it as a highly suitable material for radiation‐resistant anticorrosion coatings. While many polymers undergo significant degradation under irradiation, particularly at low dose rates and in the presence of oxygen, crosslinked lignin maintains its structural integrity with minimal changes (**Figure** **8**).

For example, cellulose exhibits a 7% reduction in breaking strength at a dose of 100 kGy, and cellulose acetate experiences a 25% decrease in shear strength at 200 kGy. Synthetic polymers like polycarbonate show a 25% drop in elongation at 100 kGy. In contrast, cured propargylated lignin coating displays only a 4% (9.5 μm coating) decrease in anticorrosion protection efficiency, even at 500 kGy in the air (Figure 7). Oxygen significantly exacerbates radiation‐induced oxidative degradation in most polymers, drastically reducing their radiation stability at low dose rates—often to just 1%–10% of their stability at high dose rates^[^ ^21^, ^36^, ^37^, ^38^, ^39^, ^40^, ^41^, ^42^, ^43^, ^44^, ^45^ ^]^ (Table S2, Supporting Information).

However, lignin's inherent resistance to oxidative processes allows it to withstand irradiation in air more effectively than other materials. This resilience is attributed to lignin's complex, aromatic‐rich, and crosslinked macromolecular structure, as well as its ability to scavenge free radicals generated during irradiation. Dominated by strong C—C (≈348 kJ mol^−1^) and aryl–ether (≈305 kJ mol^−1^) linkages, lignin is more resilient than polysaccharides, which are held together by weaker glycosidic bonds (≈240 kJ mol^−1^). Upon exposure to ionizing radiation, the delocalized π‐electron system in lignin dissipates energy via nondestructive transitions, while phenolic moieties scavenge reactive species and suppress oxidative chain cleavage. Unlike polysaccharides (chitosan, cellulose, pectin), which lack aromaticity and radical stabilization capacity, crosslinked Pro‐lignin's irregular, 3D architecture confines radical migration and minimizes long‐range degradation. Furthermore, oxygen‐induced depolymerization is largely suppressed, highlighting lignin's suitability for protective coatings in oxidative and high‐radiation environments.

Large‐scale industrial coatings have acceptable prices ranging from 1 to 10 USD m^−2^^[^ ^46^, ^47^, ^48^, ^49^, ^50^ ^]^ (Table S3, Supporting Information). The lignin‐based coating developed in this study costs ≈4600 USD m^−2^, based on laboratory synthesis and application conditions (cost analysis provided in Table S4–S6, Supporting Information). The production cost can be optimized by considering more efficient propargylation methods and minimizing the use of organic solvents. However, optimizing the synthesis was beyond the scope of this work, as it was primarily focused on demonstrating the proof of concept. Work^[^ ^51^ ^]^ provides a techno‐economic assessment of using lignin in coating production and suggests its potential feasibility, and we remain optimistic about using lignin in coating applications.

Consequently, crosslinked lignin‐based coatings offer a promising niche solution for applications requiring long‐term radiation stability in oxidative environments, outperforming conventional materials under similar conditions.

## 3 Conclusion

Modifying lignin with a propargyl moiety enables the creation of multifunctional thermosetting materials to protect metal surfaces. When combined with epoxy soybean oil as a wet strength and softening additive, lignin‐based coatings protect copper against salt and acid media. In addition to their high anticorrosion efficiency, these coatings retain their properties even after high‐dose irradiation with beta particles. This opens the way for fully biobased and highly efficient anticorrosion coatings for metal protection under challenging conditions that involve high temperature, acidity, salinity, and high radiation loading or their combinations.

## 4 Experimental Section

4.1

4.1.1

##### Materials and Methods

Softwood kraft lignin (SKL, Biopiva, Finland), propargyl bromide (Sigma‐Aldrich), pyridine (Acros Organics), dimethylformamide, DMF (Sigma), PEG‐400 (Sigma), soybean epoxy oil (Spectrum Chemical), sodium chloride, sulphuric acid (Fisher), Cu metal sheets, acetone, and ethanol (Fisher) were used.

*Cu surface preparation*: Copper sheets were cut with scissors, washed with acetone and ethanol (95%), and sanded with silicon carbide sandpaper (grade P800). Then, they were washed with hot water (45 °C), followed by ethanol and acetone, and air‐dried for 30 min. Thus, the prepared copper sheets were carefully handled using protective gloves to avoid contamination.

*Propargylation of the lignin*: In a round‐bottom flask, 0.5 g of dried lignin powder was dissolved in 35 mL of THF. After lignin dissolution, 260 μL of pyridine was added, and the solution was stirred for 15 min at 85 °C. Then, 260 μL of the propargyl bromide solution was added dropwise to the reaction mixture. After completion of the reaction (c.a. 2 h), modified lignin precipitated from the solution. The solution was allowed to cool down, and the THF was evaporated under reduced pressure. Precipitated lignin powder was washed 3× times with 100 mL of DI water and then 2× times with hexane. Lignin was then redispersed in acetone and dried at 40 °C overnight.

*Nuclear magnetic resonance (NMR)*: Quantitative analysis of hydroxyl groups of lignin was conducted using ^31^P NMR spectroscopy.^[^ ^52^ ^]^ Briefly, a modified lignin sample (30 mg) was phosphitylated with 2‐chloro‐4,4,5,5‐tetramethyl‐1,3,2‐dioxaphospholane (0.9 mmol) in the presence of *N*‐hydroxy‐5‐norbornene‐2,3‐dicarboxylic acid imine (0.010 mmol) as an internal standard and chromium(III) acetylacetonate as a relaxation agent. The P NMR experiments (256 scans, 10 s relaxation delay) were performed with a 90° pulse angle and inverse gated proton decoupling. The ^1^H NMR was recorded using deuterated dimethyl sulfoxide (DMSO).

*FTIR*: The (IR) absorbance of samples was measured using an attenuated total reflection‐FTIR (ATR‐FTIR) in the 450–4000 cm^−1^ range, a Varian 610‐IR FT‐IR spectrometer.

*Size‐exclusion chromatography (SEC)*: 5 mg of lignin was dissolved in DMSO/LiBr and filtered using a 0.2 μm syringe filter (PTFE). For measuring the molecular weight of cured lignin, propargylated lignin dissolved in DMSO/LiBr was heated for one hour at 105 °C and then filtered using a 0.2 μm syringe filter (PTFE). The cooled filtrate was injected into the SEC system.

*SEM*: SEM was performed using the JEOL‐7000 instrument (JEOL, Japan). Before use, the lignin‐coated copper sample was vacuum‐dried for 4 h at 135 °C to evaporate DMF traces.

*TGA*: 5–10 mg of lignin sample was placed into an alumina cup and heated in a dynamic regime from 25 to 800 °C in an air atmosphere with a 50 mL min^−1^ gas flow.

*Curing of propargylated lignin over the Cu metal surface*: Lignin was dissolved in DMF at a 200 mg mL^−1^ concentration and used as a stock solution. To facilitate the dissolution of lignin in DMF, a solution was heated at 50 °C for 1 h. For coating the copper, 1 mL of the stock solution was diluted with 4 mL of DMF and deposited over a 1 × 2 cm copper plate in an aluminum dish. The dish was placed into the oven preheated to 150 °C and left there for 4 h at 150 °C.

*Pro‐lignin coating with soybean epoxy oil (4.76 wt%)*: 5.6 μL of soybean epoxy oil in DMF (250 mg mL^−1^) was added to 0.56 mL of propargylated lignin in DMF (50 mg mL^−1^), followed by 1.7 mL of DMF, and cured at 150°C (sample *lignin 1x)*. For the coated samples *lignin 2x* and *lignin 4x*, the applied solution was doubled and quadrupled, respectively.

*Pro‐lignin coating with PEG (10 wt%)*: To 200 μL of Pro‐lignin (50 mg mL^−1^), 10 μL of PEG‐400 in DMF (100 mg mL^−1^) were added and heated at 150 °C for 4 h.

*Irradiation with beta particles*: The Cu samples coated with propargylated lignin were irradiated under an electron beam by a pulsed electron accelerator “Electronic” (E_e_ = 4 MeV) with a dose of 500 kGy. The electron flux intensity, I, was 0.2 μA cm^−2^. Electron fluence, Ф, was 2 × 10^15^ cm^−2^.

*Electrochemical corrosion tests*: *Corrosion solutions.* The 0.5 M H_2_SO_4_ solution was prepared by diluting 98% H_2_SO_4_ (Merck, analytical grade) with distilled water. 3.5% NaCl solution was prepared by dissolving NaCl (Merck, ≥99.0%) in distilled water. The copper specimens with deposited protective films were immersed in corrosive solutions for 24 h. Afterward, the corrosion experiments were performed.

*Electrochemical measurements.* Electrochemical tests were carried out in a conventional three‐electrode cell with EmStat 4S (PalmSens BV, Netherlands). Platinum wire and a silver–silver chloride electrode (3 mol L^−1^ KCl) were used as the auxiliary and reference electrodes, respectively. All the potentials are referred to as Ag/AgCl (3M KCl). The copper specimens with and without protective films were applied as working electrodes (working area of 0.664 cm^−2^). All measurements were performed in naturally aerated solutions at room temperature (20 °C).

Working electrodes were immersed in corrosion solutions at the open circuit potential (OCP) for 30 min to reach a stable state. The polarization resistance tests were recorded at a scan rate of 0.5 mV s^−1^ in the potential range ±250 mV versus OCP. Each test was repeated three times on different specimens to check the reproducibility. No significant deviations were observed in the solution with the same corrosive component.

The corrosion rate (*CR*, μmpy) and protected efficiency (η, %) were calculated according to the standard practice described by the ASTM Standard G 102 using the following equations:

(1) $$CR=\frac{i_{\text{corr}}\times K\times EW}{d\times S}\times 1000$$

(2) $$\eta =\frac{CR(\text{bare})-CR(\text{prot})}{CR(\text{bare})}\times 100\%$$

where *i*_corr_ is corrosion current (A), EW is equivalent weight (g mol^−1^), d is the density (g cm^−3^), S is the electrode area (cm^2^), K is a constant defined by the ASTM, 3272 mm A^−1^ cm^−1^ year^−1^.

WCA was measured with KRÜSS instruments Drop Shape Analyzer—DSA25, 2 μL water drop was used. All the images were processed with the ADVANCE software.

*Adhesion to the surface* was evaluated via a pull‐off test performed on an Instron 5960 series tensile tester (Instron, USA) equipped with Bluehill software. Circular aluminum dollies (diameter: 10 mm or 4 mm) were bonded to the coated surfaces using a two‐part epoxy adhesive and cured at 80 °C for 1.5 h. After curing, a perpendicular tensile force was applied at a constant crosshead speed of 1 mm min^−1^ until failure occurred. The maximum force at detachment was recorded, and the adhesion strength was calculated by dividing this force by the contact area, based on the dolly diameter. All samples were measured at least in triplicate. All measurements were carried out at 21°C and 50% relative humidity (RH). The failure surfaces were examined visually to distinguish adhesive from cohesive failure modes.

XPS was performed using a PHI Quantera II Scanning XPS Microprobe (Physical Electronics). The high‐resolution spectra were collected with an X‐ray source of monochromatized Al Kα operated at 25.4 W and the passing energy of the hemispheric analyzer at 50 eV. An ion gun performed the surface charge compensation. The results were analyzed using MultiPak software (Physical Electronics). The spectral binding energy scale was calibrated taking the C 1s peak at 284.8 eV as a reference. Gauss–Lorentz peak profiles (90% Gauss) were used for spectral deconvolution.

## Conflict of Interest

The authors declare no conflict of interest.

## Supporting information

Supplementary Material

## Data Availability

The data that support the findings of this study are available in the Supporting Information of this article.
