# Supplementary material for: Beta‐Radiation‐Resistant Anticorrosion Coatings Based on Lignin
Source: Small Sci. 2025 Jun 30;5(9):2500007. doi: 10.1002/smsc.202500007 (PMC12412574; doi:10.1002/smsc.202500007)
Supplement: Supplementary file 1 — Supplementary Material [file SMSC-5-2500007-s001.pdf]

Supporting Information

Beta-radiation-resistant anticorrosion coatings based on lignin

Ievgen Pylypchuk[a]*, *Oleg Tkachenko*[b], *Tetyana Budnyak*[b,c,d], *and Mika Sipponen*[a,e]*

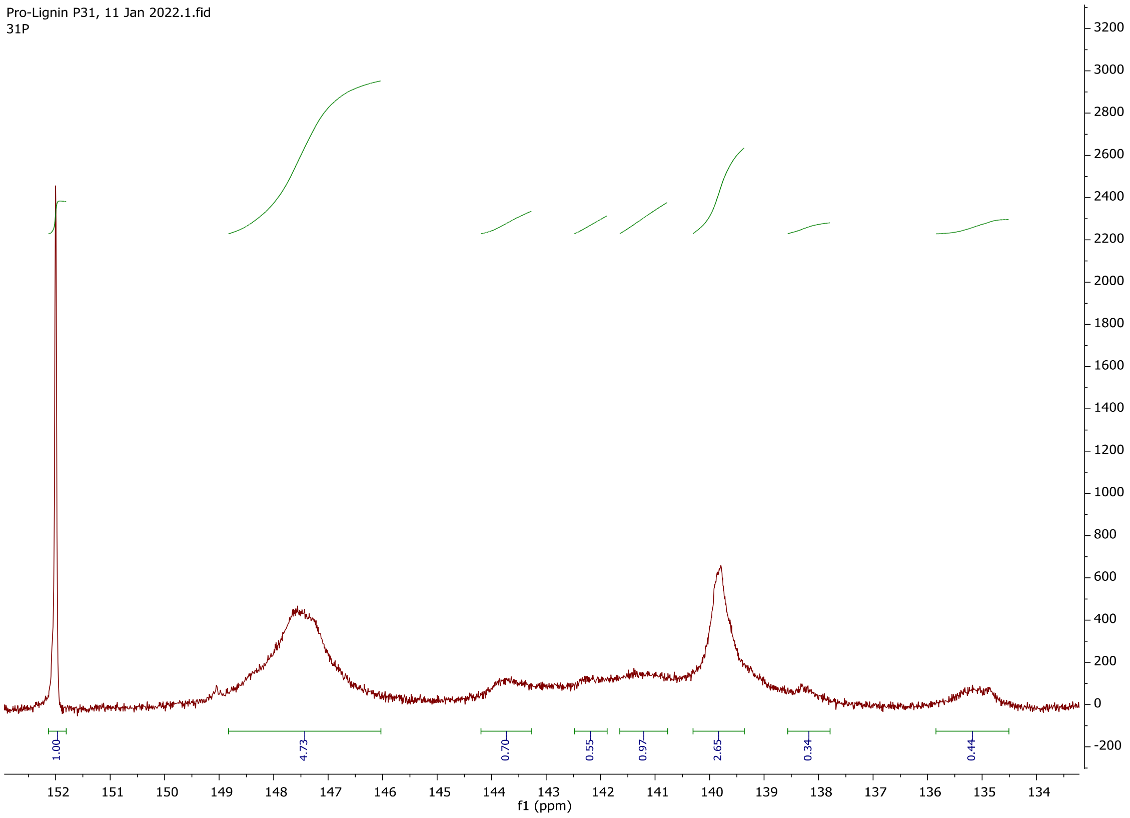

Figure S1. ^{31}P NMR data of propargylated lignin.

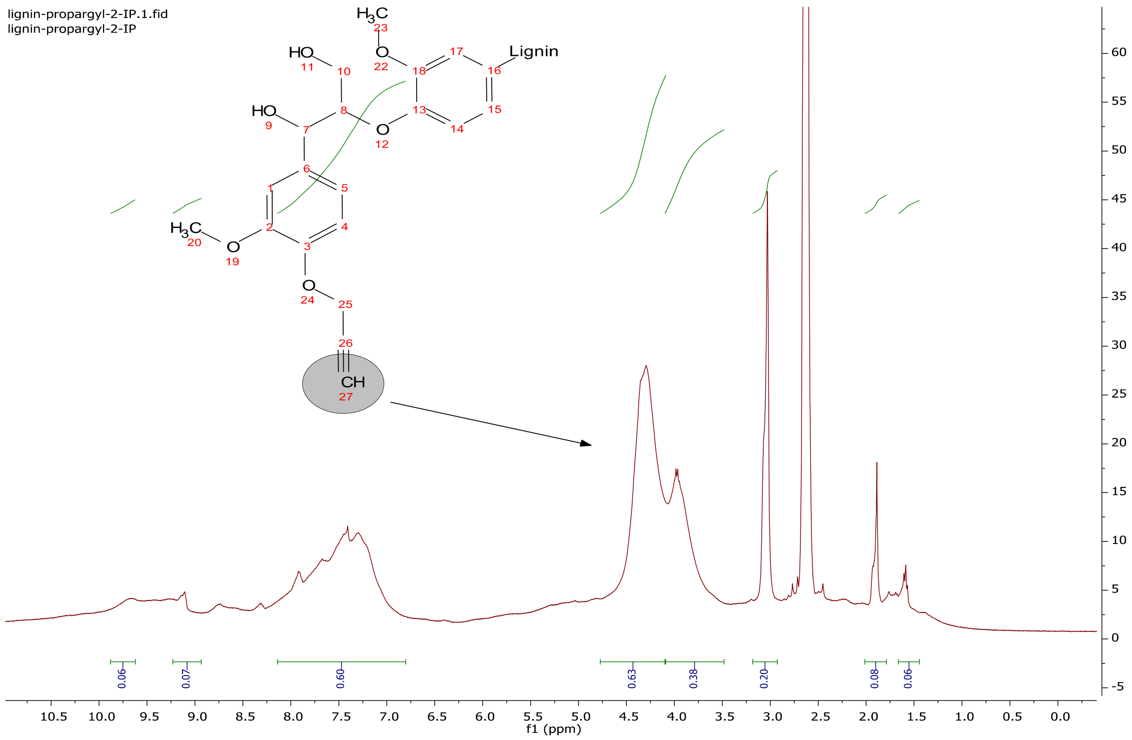

Figure S2. 1H NMR data of propargylated lignin

Table S1. The corrosion parameters were calculated by using the polarization technique for the corrosion of bare and protected copper electrodes in 3.5 % NaCl and 0.5 M H_2SO_4 at 20 °C

Coating	Corrosive system	E_{corr} (mV)	i_{corr} per surface area (µA cm^{-2})	CR, µmpy	η, %
Bare Cu	3.5 % NaCl	-0.259	9.08	111	---
Pro-lig (10 µm)		-0.139	3.84	45.3	59.6
Pro-lig epoxy (9.5 µm)		-0.177	1.30	15.8	85.7
Pro-lig epoxy (61 µm)		-0.062	2.13 10^{-2}	0.26	99.77
Pro-lig epoxy (9.5 µm) after 500 kGy radiation exposure		-0.163	1.54	18.8	83.1
Pro-lig epoxy (61 µm) after 500 kGy radiation exposure		-0.116	5.49 10^{-3}	6.69 10^{-2}	99.94
Bare Cu	0.5 M H_2SO_4	-0.043	6.7	81	---
Pro-lig epoxy (9.5 µm)		0.044	8.82 10^{-1}	11.0	86.4
Pro-lig epoxy (61 µm)		0.181	2.55 10^{-2}	0.31	99.6
Pro-lig epoxy (9.5 µm) after 500 kGy radiation exposure		0.069	1.14	14.0	82.7
Pro-lig epoxy (61 µm) after 500 kGy radiation exposure		0.034	4.6 10^{-2}	0.56	99.3

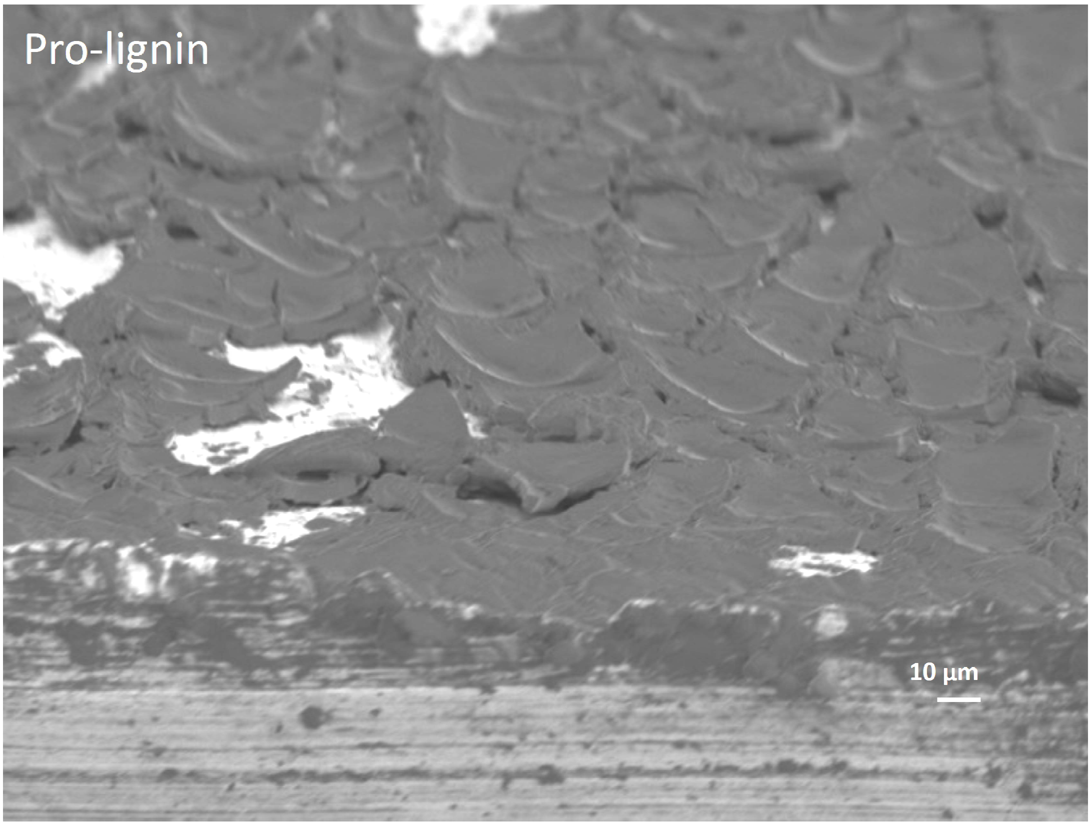

Figure S3. SEM micrograph of the copper coated with propargylated lignin coating.

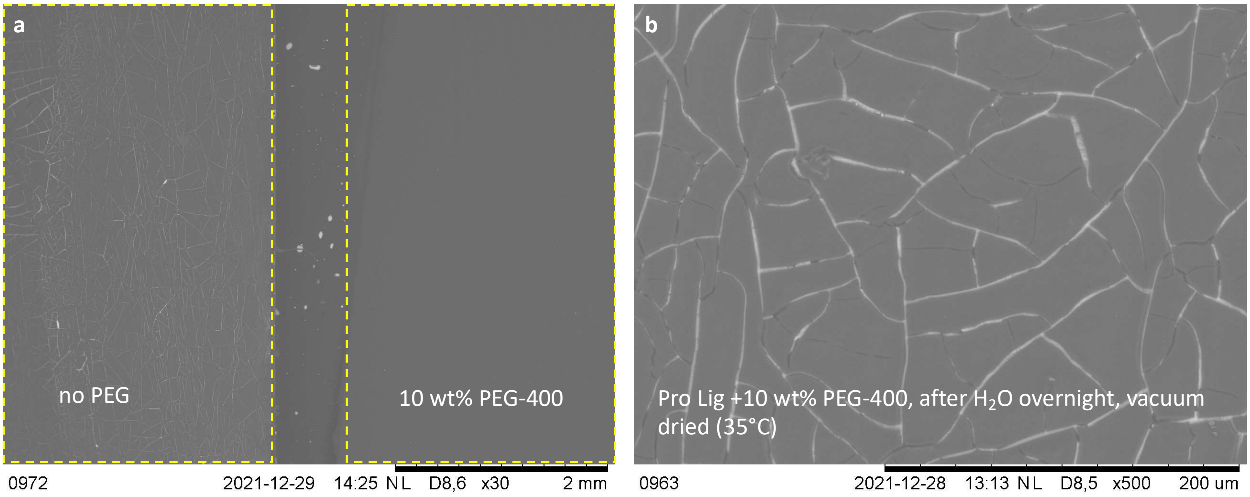

Figure S4. SEM micrographs of PEG-400 plastisized Pro-lignin coating (a) and same coating after immersed in water overnight (b).

To overcome the brittleness PEG-400 as a plastisizer was added at 10 wt.% to the curing composition. According to electron micrographs, the addition of PEG-400 allows for avoiding cracks formation (Figure 4a). It seems, that PEG-400 acts as a low-Mw plasticizer for the composition. However, the electrochemical tests were inconsistent, especially after overnight experiments (results not shown). Thus, the model test, in which PEG-containing coating was placed into DI water overnight. Electron microscopy images of such a coating before and after being immersed in water are presented in Figure 4b. PEG-400 made the coating visible smoother and avoided cracks in the dry state; However, since no covalent bonds were formed between the propargylated lignin and plasticizer, water immersion caused washing out of PEG-400. This leaching of the plasticizer led to the formation of cracks in the dried coating.

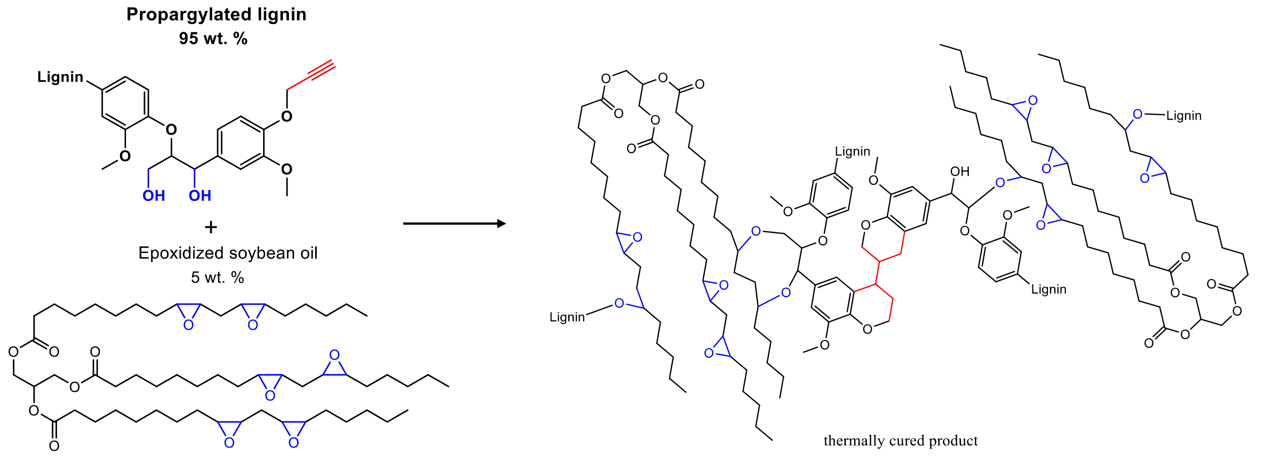

Figure S5. Scheme for thermal curing reactions occurring between soybean epoxy oil and Pro-lignin

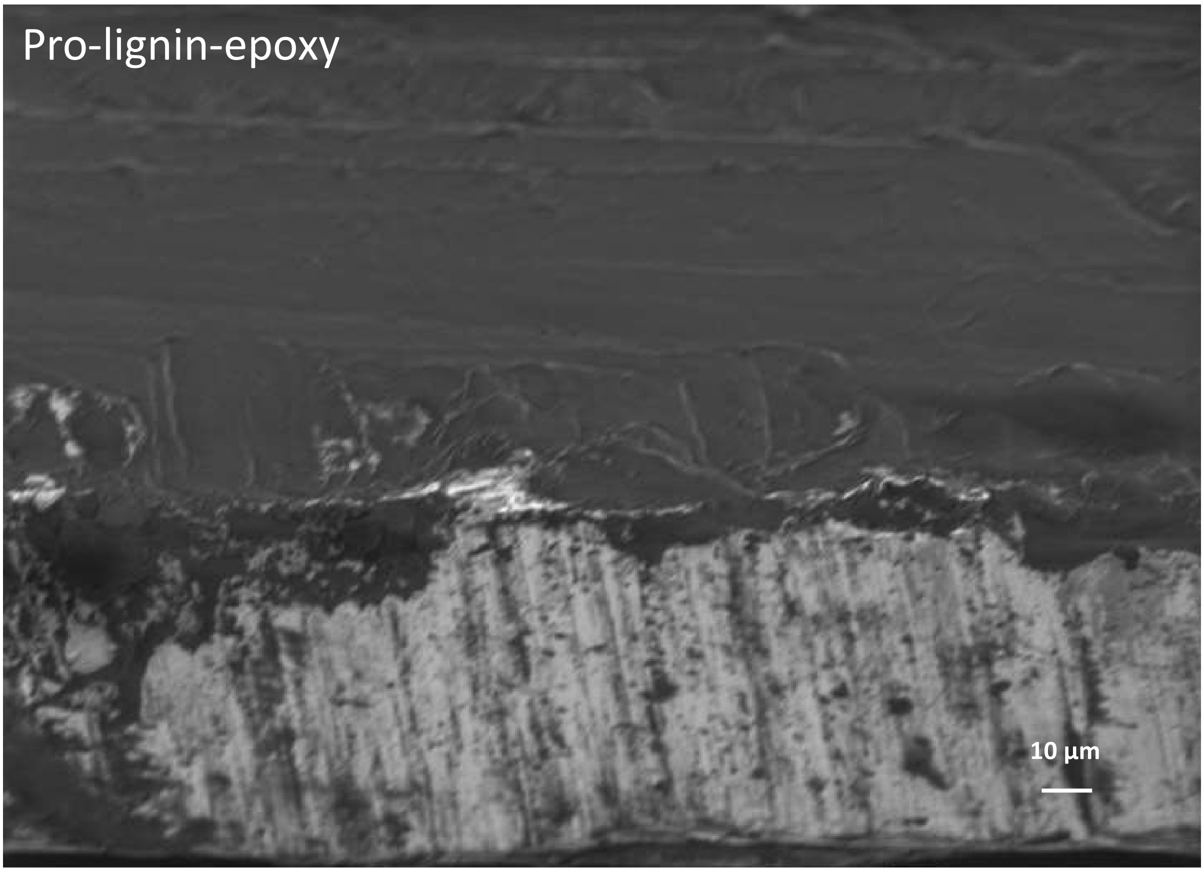

Figure S6. SEM micrograph of the copper coated with propargylated lignin epoxy coating after soaking in water for 24h.

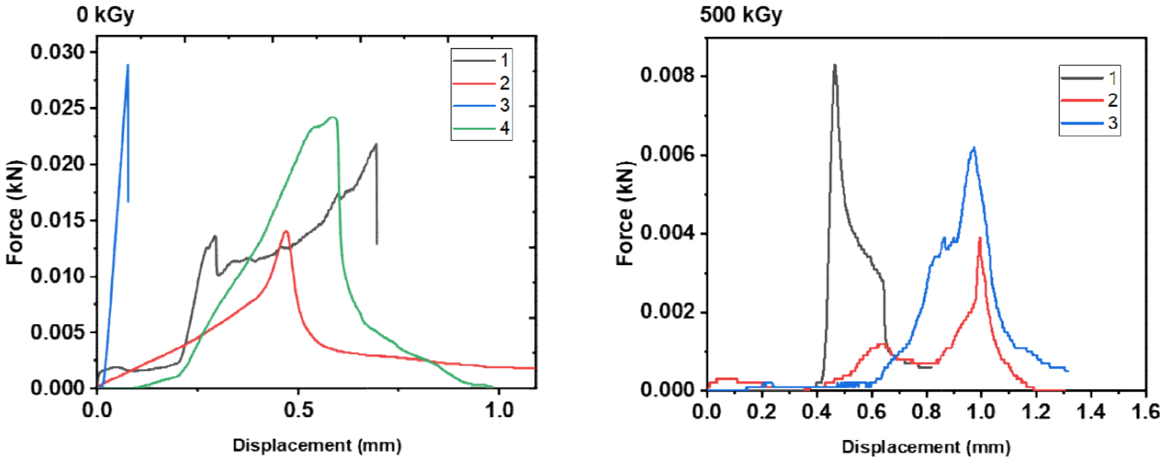

Figure S7. The pull-off test measures adhesion to the metal surface.

Radiation stability of materials in air

"It is the widespread opinion that the radiation-induced change of polymer properties depends only on the total absorbed dose. However, this is true only for irradiations carried out under the exclusion of oxygen. This means in practice high dose rates in air and thick samples. If the irradiation is carried out at low dose rates in air, the effects are strongly dependend on the dose rate. In case of thermoplastics the radiation stability at low dose rates may be only 1 to 10 % of the stability measured at high dose rates"[32].

"There is no difference in the change of properties of plastic materials with respect to the radiation effect between Electron, X-ray and gamma radiation, but there is an indirect difference between the electrons and the gamma radiation; at the radiation an oxidative decline takes place. ***For the electron radiation it is many times higher than the gamma radiation, at the same dose****"*[33].

Table S2. Radiation resistance of different materials in air.

Material	Degree of property drop, %	Change in properties radiation thershold, kGy	Property measured	Source
Cellulose	7	100	breaking strength	[42]
Cellulose acetate	25	200	shear strength	[42]
Cellulose nitrate	25	5	elongation	[42]
Ethyl cellulose	25	400	elongation	[42]
Chitosan	5	10	fraction of a chain brake (Mw, viscosity (depolymerization))	[43]
Pectin	20	0.5	Ca-pectate value (Viscosity increase (crosslinking))	[44]
Lignin	1.6	200	methoxy group content	[29]
Alginate		20–100	Mw, viscosity (depolymerisation)	[45]

Organic Solar Cells	20	6	initial photovoltaic efficiency	[46]
Casein resin	50	300	impact strengh	[42]
Polyimide	8	100	flexural strength	[47]
Polyamide	5	25	tensile modulus and yield stress	[47]
Polycarbonate	25	100	elongation	[48]
Silk fibroin (keratin)	22	375	Tensile strength 22% drop	[49]
Wool	20	400	Elongation drop by 20 % (however, no tensile strength drop)	[50]
PEEK	4.7	300	4.7% drop in Young's modulus	[51]

Table S3. Price comparison for the meal coatings.

Coating Type	Approx. Price (USD/m²)	Application	Source
Epoxy (2K) – solvent-free industrial epoxy	~$4–5 /m²	Industrial floors, tank exteriors, steel	[52]
Polyurethane (2K) – aliphatic topcoat	~$2–10 /m²	High-performance floors, tanks, roofs	[39]
Powder coatings – epoxy/polyester powders	~$0.5–1.0 /m²	Appliance, auto, structural steel	[53]
Bio-based epoxy – plant-oil 2K primer	~$3–4 /m² (est.)	Industrial floors, primers	[54]
Bio-based polyurethane – “green” PU	~$2 /m² (est.)	Roofs, floors, metal protection	[55]
Radiation-resistant biobased	n/a (no products)	–	No commercial examples found
Radiation-resistant (specialty) – phenolic/ceramic epoxies	~$10–20 /m² (high-end)	Nuclear vessels, aerospace structures	[56]
**Radiation-resistant biobased**	**4600**		**This work**

Cost analysis for production at a lab scale

Table S4. Cost of Propargylation (per 1 g Batch)

Material	Amount (for 1 g lignin)	Unit Cost (USD)	Cost (USD)	Notes
Dried Lignin Powder	1.0 g (0.001 kg)	5 USD/kg	0.005	Base material
Tetrahydrofuran (THF)	70 ml	200 USD/L	14.00	Reaction solvent
Pyridine	520 µL (0.00052 L)	300 USD/L	0.156	Catalyst
Propargyl Bromide (80%)	520 µL (0.00052 L)	1000 USD/L	0.52	Reagent
Subtotal – Materials	—	—	14.681	
THF Evaporation (0.2 kWh)	—	0.15 USD/kWh	0.03	Rotary evaporator
Drying (6 kWh)	—	0.15 USD/kWh	0.90	Oven drying
THF Recovery (70 ml)	—	0.5 USD/L	0.035	Solvent reuse
Hexane Recovery (400 ml)	—	0.5 USD/L	0.20	Solvent reuse
Acetone Recovery (100 ml)	—	0.5 USD/L	0.05	Solvent reuse
Subtotal – Energy/Recovery	—	—	1.215	
Total Propargylation Cost (1 g)	—	—	$15.90	Final cost per 1 g pro-lignin

Table S5. Coating Cost per Sample

Component	Amount / Sample	Cost (USD)	Notes
Propargylated Lignin	112 mg (0.112 g)	1.788	$15.90/g × 0.112 g
Soybean Epoxy Oil in DMF	5.6 mg	0.00092	Scaled from previous
DMF (in all solutions)	~9.04 ml	1.27	$141/L × 0.00904 L
Raw Materials Subtotal	—	3.06	
Curing (oven, 1 kWh)	—	0.15	Estimated energy per sample
DMF Recovery (~9.04 ml)	—	0.0045	0.5 USD/L recovery
Energy/Recovery Subtotal	—	0.1545	
Total Cost per Sample (4×)	—	$3.21	

Each 4x-coated sample:

- Thickness: 61 µm (0.000061 m)
- Dimensions: 2 × 3.5 cm = 7 cm² = 0.0007 m²

1 m² requires ~**1428.57** such samples

Table S6. Cost of Producing 1 m² Coating (4x Thickness)

Parameter	Value
Sample Area	2 cm × 3.5 cm = 7 cm²
Area in m²	0.0007 m²
Samples per 1 m²	1 m² ÷ 0.0007 m² = 1428.57
Cost per Sample (4× coating)	$3.21
Total Cost per 1 m² (4x)	$4587.68

References

(1) 张望杨松林. High-Temperature-Resistant, Anti-Corrosion and Thermal-Insulation Coating and Preparation Technology Thereof. CN104673063B, March 11, 2015.

(2) 郭辉李君雅陈玉. Anti-Corrosion and Radiation-Resistant Polyurea Coating for Nuclear Power Station and Preparation Method Thereof. CN113355013A, July 29, 2021.

(3) Corni, I.; Neumann, N.; Eifler, D.; Boccaccini, A. R. Polyetheretherketone (PEEK) Coatings on Stainless Steel by Electrophoretic Deposition. *Adv Eng Mater* **2008**, *10* (6), 559–564. https://doi.org/10.1002/ADEM.200800010.

(4) González-García, Y.; González, S.; Souto, R. M. Electrochemical and Structural Properties of a Polyurethane Coating on Steel Substrates for Corrosion Protection. *Corros Sci* **2007**, *49* (9), 3514–3526. https://doi.org/10.1016/J.CORSCI.2007.03.018.

(5) Singh, P.; Rana, A.; Karak, N.; Kumar, I.; Rana, S.; Kumar, P. Sustainable Smart Anti-Corrosion Coating Materials Derived from Vegetable Oil Derivatives: A Review. *RSC Adv* **2023**, *13* (6), 3910–3941. https://doi.org/10.1039/D2RA07825B.

(6) Cao, Y.; Liu, Z.; Zheng, B.; Ou, R.; Fan, Q.; Li, L.; Guo, C.; Liu, T.; Wang, Q. Synthesis of Lignin-Based Polyols via Thiol-Ene Chemistry for High-Performance Polyurethane Anticorrosive Coating. *Compos B Eng* **2020**, *200*, 108295. https://doi.org/10.1016/J.COMPOSITESB.2020.108295.

(7) Budnyak, T.; Slabon, A.; ChemSusChem, M. S.-; 2020, undefined. Lignin–Inorganic Interfaces: Chemistry and Applications from Adsorbents to Catalysts and Energy Storage Materials. *Wiley Online Library* **2020**, *13* (17), 4344–4355. https://doi.org/10.1002/cssc.202000216.

(8) Moreno, A.; Horizons, M. S.-M.; 2020, undefined. Lignin-Based Smart Materials: A Roadmap to Processing and Synthesis for Current and Future Applications. *pubs.rsc.org*.

(9) Ferruti, F.; Pylypchuk, I.; Zoia, L.; Lange, H.; Orlandi, M.; Moreno, A.; Sipponen, M. H. Recombinatorial Approach for the Formation of Surface-Functionalised Alkaline-Stable Lignin Nanoparticles and Adhesives. *Green Chemistry* **2023**, *25* (2), 639–649. https://doi.org/10.1039/D2GC03406A.

(10) Morsali, M.; Moreno, A.; Loukovitou, A.; Pylypchuk, I.; Sipponen, M. H. Stabilized Lignin Nanoparticles for Versatile Hybrid and Functional Nanomaterials. *Biomacromolecules* **2022**, *23* (11), 4597–4606. https://doi.org/10.1021/ACS.BIOMAC.2C00840/ASSET/IMAGES/LARGE/BM2C00840_0006.JPEG.

(11) Moreno, A.; Liu, J.; Gueret, R.; Hadi, S. E.; Bergström, L.; Slabon, A.; Sipponen, M. H. Unravelling the Hydration Barrier of Lignin Oleate Nanoparticles for Acid-and Base-Catalyzed Functionalization in Dispersion State. *Angewandte Chemie International Edition* **2021**, *60* (38), 20897–20905.

(12) Dastpak, A.; Yliniemi, K.; Monteiro, M. C. de O.; Höhn, S.; Virtanen, S.; Lundström, M.; Wilson, B. P. From Waste to Valuable Resource: Lignin as a Sustainable Anti-Corrosion Coating. *Coatings 2018, Vol. 8, Page 454* **2018**, *8* (12), 454. https://doi.org/10.3390/COATINGS8120454.

(13) Ren, Y.; Luo, Y.; Zhang, K.; Zhu, G.; Tan, X. Lignin Terpolymer for Corrosion Inhibition of Mild Steel in 10% Hydrochloric Acid Medium. *Corros Sci* **2008**, *50* (11), 3147–3153. https://doi.org/10.1016/J.CORSCI.2008.08.019.

(14) Abu-Dalo, M. A.; Al-Rawashdeh, N. A. F.; Ababneh, A. Evaluating the Performance of Sulfonated Kraft Lignin Agent as Corrosion Inhibitor for Iron-Based Materials in Water Distribution Systems. *Desalination* **2013**, *313*, 105–114. https://doi.org/10.1016/J.DESAL.2012.12.007.

(15) Wang, X.; Leng, W.; Nayanathara, R. M. O.; Caldona, E. B.; Liu, L.; Chen, L.; Advincula, R. C.; Zhang, Z.; Zhang, X. Anticorrosive Epoxy Coatings from Direct Epoxidation of Bioethanol Fractionated Lignin. *Int J Biol Macromol* **2022**. https://doi.org/10.1016/J.IJBIOMAC.2022.08.177.

(16) Idumah, C. I.; Obele, C. M.; Emmanuel, E. O.; Hassan, A. Recently Emerging Nanotechnological Advancements in Polymer Nanocomposite Coatings for Anti-Corrosion, Anti-Fouling and Self-Healing. *Surfaces and Interfaces* **2020**, *21*, 100734–100734. https://doi.org/10.1016/J.SURFIN.2020.100734.

(17) Zhu, Q.; Chua, M. H.; Ong, P. J.; Cheng Lee, J. J.; Le Osmund Chin, K.; Wang, S.; Kai, D.; Ji, R.; Kong, J.; Dong, Z.; Xu, J.; Loh, X. J. Recent Advances in Nanotechnology-Based Functional Coatings for the Built Environment. *Mater Today Adv* **2022**, *15*. https://doi.org/10.1016/J.MTADV.2022.100270.

(18) Vitry, V.; Hastir, J.; Mégret, A.; Yazdani, S.; Yunacti, M.; Bonin, L. Recent Advances in Electroless Nickel-boron Coatings. *Surf Coat Technol* **2022**, *429*. https://doi.org/10.1016/J.SURFCOAT.2021.127937.

(19) Nozawa, T.; Hinoki, T.; Hasegawa, A.; Kohyama, A.; Katoh, Y.; Snead, L. L.; Henager, C. H.; Hegeman, J. B. J. Recent Advances and Issues in Development of Silicon Carbide Composites for Fusion Applications. *Journal of Nuclear Materials* **2009**, *386–388* (C), 622–627. https://doi.org/10.1016/J.JNUCMAT.2008.12.305.

(20) Abu-Thabit, N. Y.; Makhlouf, A. S. H. Recent Advances in Nanocomposite Coatings for Corrosion Protection Applications. *Handbook of Nanoceramic and Nanocomposite Coatings and Materials* **2015**, 515–549. https://doi.org/10.1016/B978-0-12-799947-0.00024-9.

(21) Melin, G.; Guitton, P.; Montron, R.; Gotter, T.; Robin, T.; Overton, B.; Morana, A.; Rizzolo, S.; Girard, S. Radiation Resistant Single-Mode Fiber With Different Coatings for Sensing in High Dose Environments. *IEEE Trans Nucl Sci* **2019**, *66* (7), 1657–1662. https://doi.org/10.1109/TNS.2018.2885820.

(22) Dizhbite, T.; Telysheva, G.; Jurkjane, V.; Viesturs, U. Characterization of the Radical Scavenging Activity of Lignins—Natural Antioxidants. *Bioresour Technol* **2004**, *95* (3), 309–317. https://doi.org/10.1016/J.BIORTECH.2004.02.024.

(23) Alonso, F.; Moglie, Y.; Radivoy, G. Copper Nanoparticles in Click Chemistry. *Acc Chem Res* **2015**, *48* (9), 2516–2528. https://doi.org/10.1021/ACS.ACCOUNTS.5B00293/ASSET/IMAGES/LARGE/AR-2015-00293F_0010.JPEG.

(24) Sen, S.; Sadeghifar, H.; Argyropoulos, D. S. Kraft Lignin Chain Extension Chemistry via Propargylation, Oxidative Coupling, and Claisen Rearrangement. *Biomacromolecules* **2013**, *14* (10), 3399–3408. https://doi.org/10.1021/BM4010172/SUPPL_FILE/BM4010172_SI_001.PDF.

(25) Barroso-Bogeat, A.; Alexandre-Franco, M.; Fernández-González, C.; Gómez-Serrano, V. FT-Ir Analysis of Pyrone and Chromene Structures in Activated Carbon. *Energy and Fuels* **2014**, *28* (6), 4096–4103. https://doi.org/10.1021/EF5004733/ASSET/IMAGES/LARGE/EF-2014-004733_0003.JPEG.

(26) Haubold, T. S.; Puchot, L.; Adjaoud, A.; Verge, P.; Koschek, K. Bio-Based Bisbenzoxazines with Flame Retardant Linker. *Polymers 2021, Vol. 13, Page 4330* **2021**, *13* (24), 4330. https://doi.org/10.3390/POLYM13244330.

(27) Kiskan, B.; Yagci, Y. Synthesis and Characterization of Thermally Curable Polyacetylenes by Polymerization of Propargyl Benzoxazine Using Rhodium Catalyst. *Polymer (Guildf)* **2008**, *49* (10), 2455–2460. https://doi.org/10.1016/J.POLYMER.2008.03.031.

(28) Yan, H.; Sun, C.; Fang, Z.; Liu, X.; Zhu, J.; Wang, H. Synthesis of an Intrinsically Flame Retardant Bio-Based Benzoxazine Resin. *Polymer (Guildf)* **2016**, *97*, 418–427. https://doi.org/10.1016/J.POLYMER.2016.05.053.

(29) Sarosi, O.; Sulaeva, I.; Fitz, E.; Sumerskii, I.; Bacher, M.; Potthast, A. Lignin Resists High-Intensity Electron Beam Irradiation. *Biomacromolecules* **2021**, *22* (10), 4365–4372. https://doi.org/10.1021/ACS.BIOMAC.1C00926.

(30) Oshima, A.; Ikeda, S.; Seguchi, T.; Tabata, Y. Improvement of Radiation Resistance for Polytetrafluoroethylene (PTFE) by Radiation Crosslinking. *Radiation Physics and Chemistry* **1997**, *49* (2), 279–284. https://doi.org/10.1016/S0969-806X(96)00138-7.

(31) Andersson, M.; Pylypchuk, I. V.; Alexakis, A. E.; Liu, L. Y.; Sipponen, M. H. Esterified Lignin Nanoparticles for Targeted Chemical Delivery in Plant Protection. *ACS Appl Mater Interfaces* **2024**, *17*. https://doi.org/10.1021/ACSAMI.4C16912/ASSET/IMAGES/LARGE/AM4C16912_0007.JPEG.

(32) Wilski, H. Radiation Stability of Polymers. *International Journal of Radiation Applications and Instrumentation. Part C. Radiation Physics and Chemistry* **1990**, *35* (1–3), 186–189. https://doi.org/10.1016/1359-0197(90)90082-S.

(33) *Resistance of plastics against high energy radiation - Polyfluor*. https://www.polyfluor.nl/en/archive/resistance-of-plastics-against-high-energy-radiation/ (accessed 2023-05-19).

(34) Shivakumar, M.; Dharmaprakash, M. S.; Manjappa, S.; Nagashree, K. L. PORTUGALIAE ELECTROCHIMICA ACTA Corrosion Inhibition Performance of Lignin Extracted from Black Liquor on Mild Steel in 0.5 M H2SO4 Acidic Media. *Portugaliae Electrochimica Acta* **2017**, *35* (6), 351–359. https://doi.org/10.4152/pea.201706351.

(35) Carlos De Haro, J.; Magagnin, L.; Turri, S.; Griffini, G. Lignin-Based Anticorrosion Coatings for the Protection of Aluminum Surfaces. *ACS Sustain Chem Eng* **2019**, *7* (6), 6213–6222. https://doi.org/10.1021/ACSSUSCHEMENG.8B06568/ASSET/IMAGES/LARGE/SC-2018-06568E_0007.JPEG.

(36) Dastpak, A.; Yliniemi, K.; Monteiro, M. C. de O.; Höhn, S.; Virtanen, S.; Lundström, M.; Wilson, B. P. From Waste to Valuable Resource: Lignin as a Sustainable Anti-Corrosion Coating. *Coatings 2018, Vol. 8, Page 454* **2018**, *8* (12), 454. https://doi.org/10.3390/COATINGS8120454.

(37) Wang, D.; Claesson, P.; Zhang, F.; Pan, J.; An, R.; Shi, Y. Recent Findings on Lignin-Based Wear and Corrosion Resistance Coatings. *Corrosion Reviews* **2024**, *43* (2), 157–174. https://doi.org/10.1515/CORRREV-2024-0087/ASSET/GRAPHIC/J_CORRREV-2024-0087_FIG_007.JPG.

(38) Aguilar-Ruiz, A. A.; Sánchez-Duarte, R. G.; Orozco-Carmona, V. M.; Devora-Isiordia, G. E.; Villegas-Peralta, Y.; Álvarez-Sánchez, J. Chitosan and Its Derivatives as a Barrier Anti-Corrosive Coating of 304 Stainless Steel against Corrosion in 3.5% Sodium Chloride Solution. *Coatings 2024, Vol. 14, Page 1244* **2024**, *14* (10), 1244. https://doi.org/10.3390/COATINGS14101244.

(39) Aigbodion, V. S.; Dinneya-Onuoha, E. Unveiling the Anti-Corrosion Properties of Zn-Eggshell Particle Composite Coatings on Mild Steel in Seawater-Simulated Solution Using Starch as a Modifier. *RSC Adv* **2024**, *14* (34), 24548–24560. https://doi.org/10.1039/D4RA04283B.

(40) Jena, G.; Anandkumar, B.; Vanithakumari, S. C.; George, R. P.; Philip, J.; Amarendra, G. Graphene Oxide-Chitosan-Silver Composite Coating on Cu-Ni Alloy with Enhanced Anticorrosive and Antibacterial Properties Suitable for Marine Applications. *Prog Org Coat* **2020**, *139*, 105444. https://doi.org/10.1016/J.PORGCOAT.2019.105444.

(41) Umoren, S. A.; AlAhmary, A. A.; Gasem, Z. M.; Solomon, M. M. Evaluation of Chitosan and Carboxymethyl Cellulose as Ecofriendly Corrosion Inhibitors for Steel. *Int J Biol Macromol* **2018**, *117*, 1017–1028. https://doi.org/10.1016/J.IJBIOMAC.2018.06.014.

(42) Bruce, M. B.; Davis, M. V. Radiation Effects on Organic Materials in Nuclear Plants. Final Report. **1981**. https://doi.org/10.2172/5591289.

(43) Ulański, P.; Rosiak 1, J. Preliminary Studies on Radiation-Induced Changes in Chitosan. *International Journal of Radiation Applications and Instrumentation. Part C. Radiation Physics and Chemistry* **1992**, *39* (1), 53–57. https://doi.org/10.1016/1359-0197(92)90171-B.

(44) KERTESZ, Z. I.; LAVIN, M.; MORGAN, B. H.; TUTTLE, L. W. Effect of Ionizing Radiations on Pectin. *Radiat Res* **1956**, *5* (4), 372–381. https://doi.org/10.2307/3570426.

(45) El-Mohdy, H. L. A. Radiation-Induced Degradation of Sodium Alginate and Its Plant Growth Promotion Effect. *Arabian Journal of Chemistry* **2017**, *10*, S431–S438. https://doi.org/10.1016/J.ARABJC.2012.10.003.

(46) Martynov, I. V.; Akkuratov, A. V.; Luchkin, S. Y.; Tsarev, S. A.; Babenko, S. D.; Petrov, V. G.; Stevenson, K. J.; Troshin, P. A. Impressive Radiation Stability of Organic Solar Cells Based on Fullerene Derivatives and Carbazole-Containing Conjugated Polymers. *ACS Appl Mater Interfaces* **2019**, *11* (24), 21741–21748. https://doi.org/10.1021/ACSAMI.9B01729/SUPPL_FILE/AM9B01729_SI_001.PDF.

(47) White, I. G. Von; Tandon, R.; Serna, L. M.; Celina, M. C.; Bernstein, R. An Overview of Basic Radiation Effects on Polymers and Glasses. September 1, 2013.

(48) Chen, J.; Czayka, M.; Uribe, R. M. Effects of Electron Beam Irradiations on the Structure and Mechanical Properties of Polycarbonate. *Radiation Physics and Chemistry* **2005**, *74* (1), 31–35. https://doi.org/10.1016/J.RADPHYSCHEM.2004.12.004.

(49) Pewlong, W.; Sudatis, B.; Takeshita, H.; Yoshii, F.; Kume, T. Radiation Degradation of Silk Protein. 2000. http://inis.iaea.org/Search/search.aspx?orig_q=RN:32004917 (accessed 2023-05-19).

(50) Porubská, M.; Hanzlíková, Z.; Braniša, J.; Kleinová, A.; Hybler, P.; Fülöp, M.; Ondruška, J.; Jomová, K. The Effect of Electron Beam on Sheep Wool. *Polym Degrad Stab* **2015**, *111*, 151–158. https://doi.org/10.1016/J.POLYMDEGRADSTAB.2014.11.009.

(51) Kornacka, E. M.; Przybytniak, G.; Nowicki, A. Radical Processes Initiated by Ionizing Radiation in PEEK and Their Macroscopic Consequences. *Polym Adv Technol* **2019**, *30* (1), 79–85. https://doi.org/10.1002/PAT.4445.

(52) *Epoxy Factory Floor Paint - Industrial Grade Two-part Epoxy – Everest Paints*. https://everestfarg.se/products/factory-floor-paint-epoxy-resin?shpxid=ea5b5ebb-6dc1-47fd-9ec5-271e1a1609dd (accessed 2025-05-07).

(53) *Epoxy Powder Coating Price, 2025 Epoxy Powder Coating Price Manufacturers & Suppliers | Made-in-China.com*. https://www.made-in-china.com/products-search/hot-china-products/Epoxy_Powder_Coating_Price.html (accessed 2025-05-07).

(54) *BioCure 1100 Primer - 100% Solids Primer - 10 Gal Kit – Concrete Decor Store*. https://concretedecorstore.com/products/biocure-1100-primer-100-solids-primer-10-gal-kit (accessed 2025-05-07).

(55) *Bio-Based Polyurethane Market Analysis, Share to 2033*. https://straitsresearch.com/report/bio-based-polyurethane-market (accessed 2025-05-07).

(56) *Gulf Coast Paint PC-517 Flake Filled Phenolic Epoxy Mastic – Southern Industrial Supply*. https://southern-industrial.com/products/gulf-coast-paint-pc-517-flake-filled-phenolic-epoxy-mastic?srsltid=AfmBOoo3NrCZQ8XKkLq016EyrnDkMefV2xPSQeWYWwmCtVObs0sH-pcz (accessed 2025-05-07).
